# Money for nothing: How firms have financed R&D-projects since the Industrial Revolution

**DOI:** 10.1016/j.respol.2013.07.017

**Published:** 2013-12

**Authors:** Gerben Bakker

**Affiliations:** a Department of Economic History, London School of Economics and Political Science, Houghton Street, London WC2A 2AE, United Kingdom; b Department of Accounting, London School of Economics and Political Science, Houghton Street, London WC2A 2AE, United Kingdom

**Keywords:** R&D-project financing-history, R&D-financing institutions, Sunk costs, Historical R&D-project cost case studies, Britain, United States

## Abstract

We investigate the long-run historical pattern of R&D-outlays by reviewing aggregate growth rates and historical cases of particular R&D projects, following the historical-institutional approach of Chandler (1962), North (1981) and Williamson (1985). We find that even the earliest R&D-projects used non-insignificant cash outlays and that until the 1970s aggregate R&D outlays grew far faster than GDP, despite five well-known challenges that implied that R&D could only be financed with cash, for which no perfect market existed: the presence of sunk costs, real uncertainty, long time lags, adverse selection, and moral hazard. We then review a wide variety of organisational forms and institutional instruments that firms historically have used to overcome these financing obstacles, and without which the enormous growth of R&D outlays since the nineteenth century would not have been possible.

## 1 Introduction

A key characteristic of large high-technology firms today is that they hold enormous amounts of cash. In 2012, for example, Apple held $121bn, Google $47bn, Facebook $11bn and Amazon $5bn in cash.^1^ These firms may have many reasons for keeping such cash piles (Myers and Majluf, 1984); we will argue that a key reason is the importance of R&D to these firms, because it does not involve any bankable collateral, has a high degree of uncertainty, and long open-ended time lags, and faces several other challenges such as adverse selection and moral hazard. Therefore R&D has to be financed with cash rather than capital.

The R&D-financing issue that these technology giants are addressing with their cash piles is a classic problem that historically all R&D-intensive firms have had to address. Nowadays, the scale of the cash piles that high-tech firms keep has reached enormous proportions. Apple's cash mountain, for example, is higher than the GDPs of tens of different nations. This paper aims to give long-run historical insight into how we got here.

We examine what R&D spending looked like in the very long run, since c. 1750 and how, given the substantial financing obstacles, firms have been able to incur large R&D outlays on particular, highly uncertain projects. In order to answer this question, we explore how we can conceptualise R&D-outlays to understand their long-run historical evolution and we investigate what insights we can get into the financial and organisational nature of R&D-outlays by looking into particular historical cases, not unlike Chandler (1962), North (1981) and Williamson (1985) did to examine, respectively, organisations, institutions, and transactions. We also aim to get comparative historical insight into the order of magnitude of the costs of these particular R&D-projects.

These research questions are worthwhile for two main reasons. First, they are important because a focus on the long run allows us to see trends and changes that are not visible in the short run. Joseph Schumpeter, for example, argued that history should be included in the training of all economists. He understood ‘economic analysis’ as a combination of history, statistics and theory, and he wrote late in his career that ‘if, starting my work in economics afresh, I were told that I could study only one of the three but could have my choice, it would be economic history that I should choose.’^2^ Innovation studies scholars such as von Tunzelmann (1995), Freeman and Soete (1997), and Freeman and Louca (2001), likewise have studied history. Historians and social scientists such as Chandler, North and Williamson use history to identify and examine organisational, institutional and transactional change that we cannot see if we only examine the short run. So if we want to get deep insight into how the arrangements for financing innovation can change and what might drive their dynamics, it does not suffice to study the period since the 1990s or even since the 1970s. We need to go further back in time.

Second, historical case studies can offer us unique insights, especially since each R&D-project is to some extent, almost per definition a unique, particular case that in many respects is incomparable with other projects. Much existing work on R&D is based on analysing large data-sets of aggregate annual R&D-outlays with econometric methods. In this paper we aim to show what additional insights we can gain by taking the project as the unit of analysis and studying particular cases in the long run, in a qualitative analytical-historical way following Chandler, North and Williamson's work on the dynamics of organisations, institutions, and transactions. These historical case studies can also give us an awareness of changes in scale, time lags and organisational forms in the long run.

In this paper the project is the unit of analysis, and not the organisation (Chandler), the institutional arrangement (North), the transaction (Williamson), or other parameters. Following Chandler's historical case study approach, these cases are particular, unique cases as such and are not meant to constitute a representative sample. Nevertheless, from these particular cases we can still make some inferences. A particular project with large cash outlays, for example, can potentially refute notions such as that large scale R&D was not done in the eighteenth century, or that firms in a particular country lacked the resources to carry out the largest-scale R&D projects (Popper, 1935).

Besides the historical case study method, we also use economic history methods to gain comparative insight into R&D expenditures over time, expressing them as GDP-deflated costs, as social opportunity costs and, finally, as a fraction of an intuitive non-R&D index-case.

The main empirical evidence we examine is from Britain and the United States since about 1750, though we like to emphasise that we do not endeavour to give a systematic comparison of R&D in those two countries, for which other papers can be consulted (see, for example, Mowery and Rosenberg, 1989; Edgerton and Horrocks, 1994). We simply use the two countries to get broader insight into the general finance mechanisms. The United States is chosen because it is the largest country in the world in GDP-terms since the early twentieth century, and Britain because it was a technological leader in many areas until the mid-twentieth century and was never occupied during the period examined, unlike Germany, France or Japan.

We do not endeavour to give a complete and encyclopaedic review of each and every organisational form and institutional instrument that firms adopted. We merely try to review informally some main forms and provide a historical meta-narrative (O’Brien, 2001). We use a holistic approach and develop a new overarching framework, showing how all elements fit together, even if individual elements of this framework have obviously been studied previously. This is a work of history that aims to engage with the economics of technical change and innovation studies (ETIS). It does not aim to be an economic or management study, and not a standard innovation studies paper either.

This paper aims to contribute to innovation studies by showing how a long-run historical perspective, following the tradition of Nick von Tunzelmann and Chris Freeman, can give us some additional insights with respect to present-day studies. We return to very basic facts about R&D. Our approach is not economic; we focus on practical problems that firms faced and show the role of market imperfections. We aim to show how the current R&D-financing framework emerged from the past and how the factors we discuss are also important for policy and practice and for future experimentation with organisational forms and institutional instruments.

What follows first reviews the most important obstacles firms encountered when they wanted to finance R&D. In the next section we first examine growth rates in the very long run to identify trends, and then several particular historical R&D-projects for which we could trace the total cash outlays. In the subsequent section we review several organisational forms and institutional instruments that firms have historically adopted to overcome the R&D-financing problem. A final section concludes.

## 2 Challenges to the finance of research

We argue that the financing of R&D is made difficult by five challenges: the presence of sunk costs, real uncertainty, long and open-ended time lags between outlays and pay-offs, adverse selection, and moral hazard. We will discuss these in turn.

### 2.1 Sunk costs

Historically, a formidable challenge for R&D-financing has been the fact that costs are sunk (Sutton, 1998). Sunk costs are costs that must be incurred to achieve a project's aim, that are incurred once, and that cannot be recovered upon exit. R&D-costs are mostly sunk: if the outlays do not lead to a marketable product, little residual value is left. Furthermore, R&D costs are incurred ‘internationally’ and do not have to be incurred again with the entry of each new market, as is the case with, for example, advertising (i.e. the results of R&D costs, the successful R&D-projects, can be marketed internationally) and the results of R&D can to some extent be protected against imitation by intellectual property and trade secret law.

The small residual value of an uncompleted R&D-project also implies that there is little collateral. Given this absence of collateral, given the absence of a cash flow from which to make regular interest payments, and given that the sum needed is not precisely known ex-ante, banks generally are unwilling to provide loans for R&D. The level of sunk R&D-costs differed between industries and varied over time (Kamien and Schwartz, 1982, p. 85).^3^ Although precise evidence is lacking, undoubtedly costs of R&D-projects increased over time and over the course of a technological trajectory. In the empirical section below we aim to gain historical understanding of the scale and growth of sunk costs in the long-run.

Technical or generic solutions to the sunk costs aspect of R&D have been developed, and most are applied nowadays by venture capital firms (Table 1). They include funding in stages, whereby initially only a limited sum is committed, until a certain milestone is reached that gives more information about the R&D-trajectory, after which a decision is made about whether to sink more money, and so on. This is not unrelated to the option approach, in which entrepreneurs see an R&D-outlay as the buying of a call option allowing them to decide at a later time whether to continue. Hartmann and Hassan (2006) provide a detailed study on the prevalence of this approach in the pharmaceutical industry.

The accounting practice of immediately writing off R&D-outlays, taking them out of existing cash flow, also mitigates the financing problems associated with sunk costs.^4^ They will not have an effect on company financial performance indicators in future years, unlike (physical) capital investments: if an R&D-project fails, few write-offs have to be made, and this may also help mitigate a sunk costs bias in decision making. Government financing of R&D-outlays is another tried and tested solution, prevalent, for example, in the defence sector.

### 2.2 Nested uncertainty

Uncertainty was an important challenge for the financing of R&D projects. Although we cannot measure it exactly historically, we can identify what factors may have increased or decreased uncertainty. R&D is almost defined by real (uninsurable) uncertainty (Knight, 1921). We identify here four types of successive real uncertainty: technical, strategic, market and profit uncertainty.^5^ Technically, it is uncertain whether R&D-outlays will lead to a working innovation, and if they do whether this innovation is what was originally specified or expected. Even if successful in technical terms, the firm faces strategic uncertainty, uncertainty depending on the actions of an intelligent opponent: are competitors doing similar research and if so, could they launch their product first? Even when these two uncertainties are resolved the firm faces market uncertainty about whether the market for the innovation remains as it was expected to be when the R&D-project commenced. And when the preceding three uncertainties have been resolved, the firm still faces profit uncertainty about whether its business model is able to capture the value of the innovation.

The problem of real, uninsurable uncertainty has been poignantly summed up by Joseph Schumpeter (1942, p. 82): “Long-range investing under rapidly changing conditions, especially under conditions that change or may change at any moment under the impact of new commodities and technologies, is like shooting at a target that is not only indistinct but moving-and moving jerkily at that.” Because of real uncertainty Keynes (1936) argued that businesspersons needed animal spirits, hunches, to take action and invest in an uncertain world (see also Freeman and Soete, 1997, pp. 242–264). Pure, extensive and complete rational calculation was impossible and led to paralysis.

Arrow (1962) identifies uncertainty also as one of the three reasons why, in his view, the allocation of resources for R&D in an economy is suboptimal at the aggregate level.^6^ Williamson (1985) identifies uncertainty as one of the key three dimensions that determine transaction costs; the other two are the frequency of transactions and asset specificity, which is the value of the underlying asset outside of the particular transactional relation. Clearly, R&D-projects score very low on all three dimensions: they are highly uncertain, the frequency of transactions is close to one, and they are highly asset specific.

Historically, some developments and institutions have mitigated real uncertainty, while others have increased it (Table 2). The net effect is unclear. Though undoubtedly scientific advances, patents and market research have all mitigated uncertainty, the constant emergence of new technological trajectories, antitrust laws and the growth of highly income-elastic products and services may have increased it. In the United States, for example, from the late nineteenth century antitrust laws increased uncertainty by preventing collusion, while the resulting merger wave reduced uncertainty by taking out and using competitors’ R&D pipelines. Some institutions worked both ways: prizes reduced profit uncertainty for the innovator by guaranteeing payment for success, and for the prize-financer by setting a maximum payment for the innovation; by contrast, they increased strategic uncertainty for innovator and prize-financer alike, by attracting many competitors to the race.

Technical solutions to the uncertainty problem included the option approach, in which R&D was seen as a process to reduce uncertainty in successive steps, or literally keep development options open in the face of competitive threats.

Collusion has sometimes been an effective means for mitigating strategic uncertainty. In interwar Switzerland, for example, the drug firms Hoffman-LaRoche and Ciba had a mutually exclusive agreement in which one focused on vitamins, the other on hormones. Edgerton (1987) argues that British firms in the interwar period often used R&D-projects as bargaining tools when negotiating with competitors. Joint R&D projects are another way to reduce strategic uncertainty. Patents, of course, reduce profit uncertainty by increasing the costs for competitors to imitate the innovator.

### 2.3 The time lag

The ‘roundaboutness’ of R&D, the time lag between outlays and eventual profits, if any, is another important challenge to the financing of R&D. We discuss it here separately because it was already identified as a key problem by economists such as Schumpeter, Frank Knight, John Maynard Keynes and John Hicks, because it is historically measurable, and, finally, because firms do take multi-stage time lags into account when making decisions about R&D-financing.

Historically speaking the time lag is important, as never before in history did firms face such long and uncertain multi-stage time lags and such roundabout production as in the period since 1750. It is a unique feature of many modern economies that firms are willing to take resources out of the immediate production process for many years, only for uncertain benefits in a distant future. One could argue that only modern society, a society with modern institutions and modern economic growth enabled private firms to deal with these enormous time lags.

R&D is characterised by a long, multi-stage time lag between cash outlays and cash flowing in (Holmstrom, 1989). This lag can be divided into the lags between the start of research and a proven invention, the proven invention and a working prototype, the first prototype and one that can be easily manufactured, the final prototype and start of production, the start of production and commencement of sales, commencement of sales and revenues coming in, and, finally, incoming revenues and profits. At each point a decision is made whether to continue, and successively more cash is needed. The exact length of these nested lags cannot be predicted in advance, and the external and internal/opportunity costs of cash may vary over these lags.

The time lag has been noted as a fundamental economic dynamic by economists such as Schumpeter, Knight, Keynes, Arthur Lewis and Hicks (von Tunzelmann, 1995, pp. 66–67). Schumpeter (1911, p. 42), for example, noted that “every period operates with goods which an earlier period prepared for it, and in every period goods are produced for use in the next.” His later observation that long-range investing is like shooting at a jerkily moving target (see the preceding section) also underlines the importance he attributes to the time lag. Likewise, Knight (1921) noted that uncertainty increased sharply as the time lag between product design, production and sales increased, and that the time lag itself was therefore an important challenge for entrepreneurs. Keynes (1936, p. 46) wrote that “the entrepreneur has to form the best expectations he can as to what the consumers will be prepared to pay when he is ready to supply them after the elapse of what can be a lengthy period; and he has no choice but to be guided by these expectations, if he is to produce at all by processes which occupy time.” Hicks (1973) likewise rejects the notion of a timeless equilibrium and distinguishes between a construction phase with no output, and an operation phase in which revenues need to cover the sacrifices of the construction phase as well as its own output. Long, open-ended time lags make firms reluctant to invest, because recalling one's money is difficult, as cash is not coming in until the end. These economists refer mainly to all fixed outlays, while for R&D the roundaboutness and thus the time lag's effects will be even more prominent.

Given the nature of R&D it is difficult to establish the direction and extent of changes in the average time lag. von Tunzelmann (1978), defining it as the time between patent application and first commercial introduction, suggests that there is no clear evidence that this time lag has been shortening since the Industrial Revolution. The time lag for steam engines and related machinery was rarely more than five to six years, much lower than for inventions with similar intensity in late nineteenth and twentieth century, according to von Tunzelmann (see also Mansfield, 1968, p. 110). What has increased is the roundaboutness of the R&D process. During the Industrial Revolution inventors and innovators often were the same persons, and this is far less likely to be the case today. Over technological trajectories time lags often increased as the ‘low-hanging fruit’ disappeared. Examples are the development of catalytic cracking (Enos, 1962) and the aircraft and pharmaceutical industries during the twentieth century.^7^

Obviously, the time lag is not fully controllable. Scherer (1967) and Kamien and Schwartz (1982, p. 132) note diminishing returns to the time compression of R&D. The more time is reduced, the higher the costs, as one cannot await the outcomes of previous experiments before proceeding with new ones. At the Edison lab in the late nineteenth century, for example, the time scale of experimentation was enormous. To make carbon filament 6000 different plant species were tried and for the nickel-iron battery 50,000 separate experiments were performed (Dodgson and Gann, 2010, p. 91).

Generic solutions for the time lag include immediate write-offs of R&D-outlays, having a clearly defined ‘green light’ point, at which a decision will be made about whether or not to sink substantial amounts of cash, and the IPO for start-up companies, which allows investors to cash in before the firm has a positive cash flow (Table 1). R&D of complex products is sometimes timed so that the critical elements and largest cash outlays are made closest to market. Japanese car makers, for example, often develop details such as rear-view mirrors and bumpers first and critical elements such as an engine last, in order to achieve the shortest time-to-market.

### 2.4 Information asymmetries

Besides the three inherent factors, two well-known transactional obstacles inhibit R&D financing as a result of information asymmetries between innovator and financer: adverse selection and moral hazard (Goodacre and Tonks, 1995; Hall, 2002).^8^ Given that historically these factors have been important, since a great deal of historical evidence can be interpreted as efforts to mitigate information asymmetries,^9^ we find it important to discuss them here.

Adverse selection involves hidden information: the financer often cannot objectively establish the likelihood of a technical venture's success because the innovator is better informed. On average, projects offered for external finance therefore have a lower probability of success. To remedy this, the financer is not likely to demand a higher stake, because purveyors of the most problematic projects would most readily accept such demands. Matters are made more difficult because generally innovators will be reluctant to disclose information (Kamien and Schwartz 1982, p. 28), and this reluctance is probably higher for better projects.

Generic solutions to adverse selection include investor initiation, in which the investor approaches entrepreneurs or firms with promising projects, rather than the other way around, a technique also commonplace today in mergers and acquisitions. Scientists on the boards of external financers, financing a specific industry, and financing with a consortium of informed investors are other tried and tested ways to reduce adverse selection.

Although historical quantitative indications of adverse selection are difficult to come by, Beatty et al. (1995) find that for Research and Development Finance Organisations (RDFOs), U.S. legal vehicles that firms can use to finance a particular, well-defined later-stage R&D-project externally, the cost of formation is two to three times that of ‘seasoned equity offerings of similar size’.^10^ This suggests very high information costs, and Beatty c.s. find that any firm with a sufficient cash flow or pile is likely to finance R&D internally and not use an RDFO.

The second information asymmetry, moral hazard, involves hidden action. Ex-post an innovator could take more risk than originally agreed and obtain larger profits if successful, while the external financiers would bear the additional risk of bankruptcy. Alternatively, the scientist might choose to maximise fame and recognition by pursuing scientifically rather than commercially interesting leads. Financers have often lacked the expertise to establish what the innovator was actually doing. Large firms and wealthy individuals could alleviate moral hazard more than others because they could invest more of their own resources in an R&D-project and so attract outside investors (Kamien and Schwartz, 1982, p. 85).

Again, since we find substantial historical evidence of firms developing ways to mitigate moral hazard, this must have been an important problem for the financing of innovations historically. For in-house research, solutions to moral hazard included individual incentives such as bonuses, combined with large fixed or group payments to ensure teamwork continued. Lab architecture has also been important. In the late nineteenth century Bayer, for example, designed a new lab architecture by arranging the chemists in workbenches laid out in a U-shaped pattern with partitions up to chemists’ shoulders (Beer, 1958). This allowed a manager to see what the chemists were doing, and communication was possible, while the researchers could still work individually and independently. This procedure prevented researchers from pursuing their own agenda or leaving the firm with hidden inventions. For external finance, generic solutions have included convertible debt, large equity stakes for the key scientists, board seats for the investors, and regular site visits.

## 3 Historical evidence

The five obstacles meant that innovators needed cash, without underlying collateral, not capital. No well-functioning market for cash with a law of one price existed. The implicit costs of cash differed widely between firms, and market interest rates and stock returns only set a floor under pay-offs investors expected from R&D-projects. Present-day empirical evidence shows that R&D-outlays are generally sensitive to cash flow or cash piles of firms (see Section 3.3).

Given that cash was crucial for R&D financing, and that it generally was tied to information, personal contacts or organisational structure when it was made available for R&D, the mechanisms that enabled the accumulation of cash and helped it being used to finance R&D were important. There simply did not exist a market mechanism that could generate cash for any R&D-project with a positive expected value. Little is actually known historically about these cash outlays, their size, their scale and how they were financed. We aim to gain historical insight by looking at long-run R&D growth rates and at historical case studies of R&D-projects in Britain and the United States.

Two key reasons why the market for R&D cash did not work were the heterogeneity of R&D-projects and the absence of perfect information. The cash could only be put out by persons with sufficient knowledge of the technology and organisation of a project at hand and this knowledge was not generally tradable. Entry and exit in R&D-projects was also complicated. Historically, scholars have argued that firms are institutions that reduce transaction costs (Coase, 1937; Williamson, 1985), mitigate bounded rationality (Simon, 1997), solve the agency problem (Jensen and Meckling, 1976; Fama, 1980), provide an incentive system (Holmstrom and Milgrom, 1994) or assign property rights efficiently (Hart, 1995). In addition, firms could be seen as institutions that can effectively allocate cash to R&D-projects, by combining it with knowledge and monitoring systems.

An evolutionary positive feedback process in which surviving organisations amassed ever larger cash flows and better knowledge was undoubtedly important for financing R&D. A long time was needed for such unique organisations to emerge and develop. It only took a few months for a large firm to go bankrupt, but many decades to rebuild the R&D-cash allocation function, including the free cash flow and knowledge, from scratch. This entropy also implied that upon dissolution of a firm, information was lost, sometimes forever, that could not be fully traded or disclosed and sometimes not even articulated. This raises intriguing dilemmas for industry policy.

### 3.1 Long-run growth rates of R&D outlays

The aggregate effect of an evolutionary cash accumulation process at work should be observable in the long-run pattern of R&D expenditure relative to GDP. Before 1900 little evidence is available on aggregate R&D expenditure, though our R&D-project case studies for Britain, below, suggest that it is unlikely that the real growth rate of R&D-expenditure was lower than real GDP-growth between the start of the Industrial Revolution and 1900.

From 1910 onwards, several estimates are available for R&D-outlays in Britain. These are not derived directly from precisely recorded figures in national accounts or censuses of industry, but are best estimates based on surrogate indicators. Given the data quality, we focus here on the *growth* rates of R&D outlays, which may be more reliable, informative and comparable, and less dependent on the particular measurement concept used, than absolute outlays. It is clear that British R&D outlays grew rapidly—almost five times as fast as GDP—in the three decades since 1910, then almost eight times as fast during the war, and then six times as fast in the immediate post-war period (Table 3).

The high multiples between the 1900s and the 1960s, of five to eight times GDP-growth, show that the economy managed to allocate ever more cash to R&D-projects with a long-run pay-off. More and more resources were taken out of direct production and put into long-term roundabout production. The economy developed and used a variety of institutions that enabled ever more cash to be sunk in R&D-projects. We will discuss these institutions in the next section.

During the 1960s, the growth of R&D outlays slowed down to about the same rate as GDP-growth, and after that both aggregate and private R&D grew substantially slower than GDP, while R&D in higher education increased with several times the rate of GDP-growth. These growth rates suggest an inverted U-shape of relative R&D-growth, with R&D growth reaching a peak during the war and slowing down subsequently. The period since the 1970s, may have been unique, because for the first time since the Industrial Revolution, the growth of R&D-outlays has no longer exceeded GDP-growth.

For the United States, we cannot reject a similar long-run pattern of R&D outlays. Although reliable estimates are missing for the period before the 1930s, the growth rate of scientists employed in corporate R&D labs suggests that R&D-outlays grew about four times faster as GDP-growth, a figure not out of line with the growth of private R&D outlays in the 1930s, and the British relative R&D-growth multiples. The estimates suggest that after the war, the relative growth rate slowed down, and that from the 1970s, aggregate U.S. R&D was growing about as fast as the economy.

These data show the enormous scale at which cash historically has been allocated to R&D-projects in spite of the major obstacles we noted. R&D outlays that grew faster than GDP also showed the increasing opportunity costs of aggregate R&D: society was willing to give up an increasing share of its current income to sink into R&D-projects with an uncertain outcome at an indefinite moment in the future.

### 3.2 Historical case studies of outlays on particular R&D projects

We also have collected historical case studies of particular R&D projects. Case studies are important in historical approaches to innovation, and in doing these we follow work done by, for example, Alfred Chandler, Douglass North and Oliver Williamson (see also the Introduction). In our case studies, unlike many other papers on R&D finance, the particular R&D-project is the unit of analysis, rather than other units such as firm R&D outlays, R&D-sales ratios, patents, etc.^11^

The collection of cases we have assembled is simply that, a collection of case studies. We do not claim that they are in any way necessarily representative, and this is difficult in R&D-projects anyway, as each R&D-project by definition was unique. We should also note that the cost data should be seen as broad estimates and may not be precisely comparable. Total project costs have been taken from the source. For many amounts it is unclear to what extent development and pilot production have been included. For some amounts, such as the spinning jenny, it is known that they are for the innovation only, for others more costs were included. In the case of the water frame, for example, the building of two pilot plants are included in direct costs, making it one of the most costly private innovations of its time. The costs in the table should therefore be read only as rough indications of the magnitude of expenditures.

The costs collected are total costs of the R&D-project in nominal pounds or dollars of the time. These costs are then converted into real amounts that are comparable over time using three different approaches. First, we correct them for price rises of all goods and services by converting the nominal amounts into constant pounds or dollars using the GDP-deflator, based on the middle year of the R&D-project's duration (Officer and Williamson, 2010).

Because the resulting real amounts are not always intuitively easy to interpret and compare, we have developed a second way to express project costs, which we call the Empire State Index. Officer and Williamson (2010) introduce the Empire State Building in New York, completed in 1931, as a good historical costing example of a non-R&D project. We therefore use its construction costs as a historical comparator. These costs may be intuitively more readily understood than the more ‘abstract’ costs of R&D-projects. The scale of the Manhattan project, for example, becomes immediately clear, as it amounted to about forty Empire State buildings, making it arguably equal to much of the construction value of real estate on Manhattan. To facilitate comparisons between Britain and the United States, the Empire State Building construction costs have been expressed in British pounds using the 1931 exchange rate, so that we also can express the value of British R&D-projects in Empire State Buildings.

A third method of comparing R&D costs is using their GDP-share. On the demand side, the GDP-share shows the opportunity costs to society; it reflects what share of income the economy needed to give up for the project (Officer and Williamson, 2010). On the supply side, this measure ‘corrects’ the costs for national market size—it expresses costs in relation to this market size, assuming that the higher the share, the more difficult to extract the finance and resources from the production process. This might be a useful heuristic tool as it links R&D costs to the capacity of a growing market to generate income that can be sunk into R&D projects. However, it is innocent of the fact that as market size grows, the R&D-costs for a given quality level remain the same, and for this purpose the GDP-deflator might be better.

Table 4 shows selected historical cases of mostly successful British innovations for which cost data have been located. Project costs and time lags varied substantially between projects. Most innovations included were product innovations, though chemical innovations often were both product and process innovations, since developing a viable manufacturing process was often as difficult as developing a compound itself. For the cases presented, cash flow financing was not that important until the late nineteenth century

These anecdotal figures suggest that the sharp growth in R&D expenditure may not be something characteristic solely of the twentieth century, but may already have started during the Industrial Revolution. For the development cost associated with artificial silk, for example, a hundred Hargreaveses could have developed the spinning jenny. If this difference was at all representative, it would point to a growth in real outlays on a major innovation of 3.6 percent per year between 1767 and 1904, compared to a GDP-growth of 2.0 percent.

Likewise, for the cost of ICI's war-time nuclear research, one could pay 26 Charles Babbages to develop a difference engine. If at all representative, this comparison points to a growth rate of real outlays on major government R&D-contracts of 2.8 percent annually between 1823 and 1941 compared to a GDP-growth of 2.0 percent.

Expressing costs as share of GDP shows project-sizes fluctuating between four orders of magnitude, from £1m for the first channel-crossing by plane to £437m for the development of semi-synthetic antibiotics. This range is probably not fully representative; the inclusion of cases from the aircraft industry and large government-funded projects would surely increase the range upwards. Yet it does help us to put historical project costs into perspective. The Board of Longitude's ship's clock project, for example, which cost £217m, had the scale of a twentieth century war time government research project, being about as large as ICI's nuclear research programme. Given that over the eighteenth century the Board of Longitude paid over seven times Harrison's amount in total for all kinds of innovations that helped establish longitude, total costs were much higher, around £1.5bn—about seven times ICI's war time nuclear R&D.

For the United States R&D-projects for which cost figures were reported have also been located (Table 5). As with the British cases, costs are probably not exactly comparable and should be seen as broad estimates. Besides varying costs, the nature of R&D projects also diverged considerably, as can be seen from the table. In catalytic cracking and aircraft R&D a ‘low-hanging fruit’ pattern is visible, with low initial but rapidly escalating R&D-costs (Enos, 1962). Trajectories seem to have followed the colloquial saying that R&D-costs must rise exponentially for a linear increase in innovation.

Cash flow was the dominant way of financing for the case studies surveyed. R&D-projects earlier than the cases in Table 5 probably used a greater variety of financing methods, as in the British case. Standard Oil of New Jersey's purchase of IG Farben's non-German patent rights was the largest pre-war project among the cases. A major advantage was, of course, that the technology had already been developed, meaning that there were fewer sunk costs (as the patents could be sold on), little uncertainty and hardly any time lag, adverse selection or moral hazard. In theory, it was also possible to use the portfolio as collateral, making financing easier. Though one could probably not group the purchase of a patent-portfolio under R&D, it did resolve most of the obstacles to financing R&D. Disadvantages were probably the high price paid, the considerable knowledge and development costs needed to use the patents, and the fact that somebody had to have already completed the necessary R&D.

Costs of R&D-projects could differ enormously, sometimes by several orders of magnitude. The R&D for cellophane or for Lockheed's Vega streamlined aircraft was only a few million dollars, measured in GDP-share, while the development of television cost over $1bn, the Manhattan Project R&D over $4bn, its pilot plants $111bn, and the manned moon landing $558bn. The Boeing 747 cost over $12bn to develop, four thousand times more than the Lockheed Vega forty years earlier, and even more if we used GDP-deflated costs. These cases show the enormous scale at which firms burnt cash on R&D-projects, in spite of the major financing obstacles.

The cases since the 1970s that were financed with venture capital show that the pre-IPO amounts sunk into these projects were not extremely big compared to R&D projects earlier in the century, or even in the eighteenth and nineteenth centuries. Key differences were that the venture capital-backed projects often developed discoveries made in the sharply growing government and university labs and that the projects did not yet have positive cash flow before IPO; the IPO itself was a way to get more cash to get the project going. It shows how the IPO could be a device to increase project-scale by requiring in the pre-IPO stage funding at a similar order of magnitude that had been used before for other R&D projects.

**Fig. 1:**
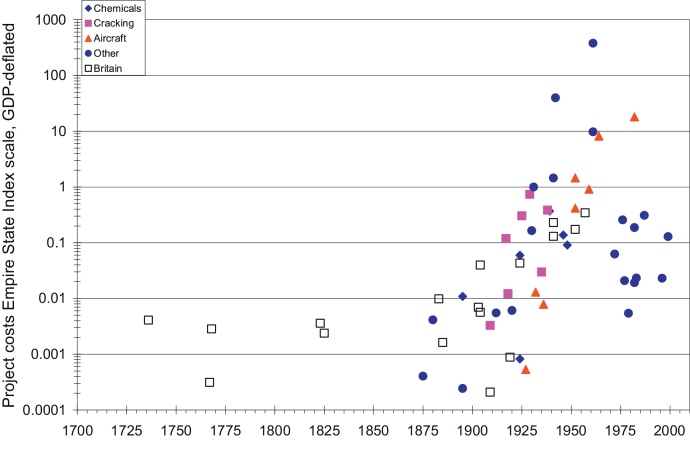
Real costs of selected historical cases of completed R&D-projects, Britain and the United States, 1700–2000, Empire State Index; semi-logarithmic scale. *Notes*: ‘Britain’ refers to the British cases from Table 4; the other labels refer to the respective categories of the U.S. cases in Table 5. The Empire State Index divides the real GDP-deflated R&D-costs by the construction costs of the Empire State Building (1931).

Sources: Tables 4 and 5.

**Fig. 2:**
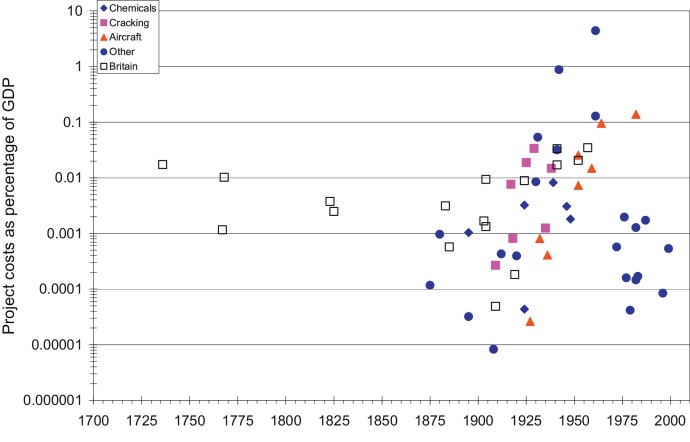
Real costs of selected historical cases of completed R&D-projects, Britain and the United States, 1700–2000, GDP-share; semi-logarithmic scale. *Notes*: see Fig. 1.

Sources: Tables 4 and 5.

**Fig. 3:**
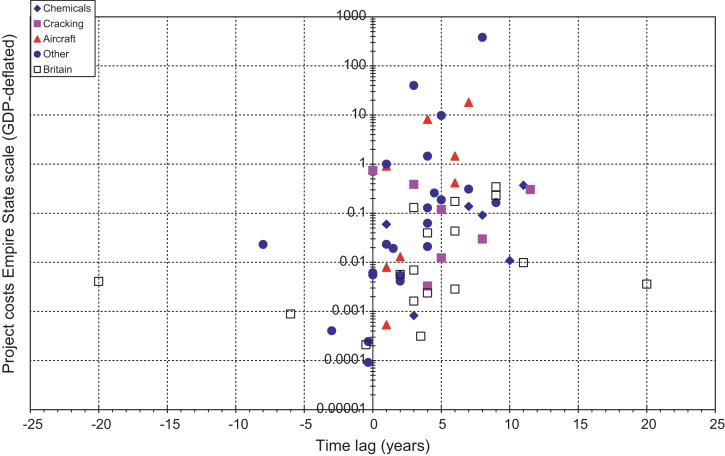
Real costs of selected historical cases of completed R&D-projects and their time lags, Britain and the United States, 1700–2000, Empire State Index and years; semi-logarithmic scale. *Notes*: see Fig. 1.

Sources: Tables 4 and 5.

The cost of the cases in both countries from Tables 4 and 5 are expressed in the Empire State Index (ESI), GDP-share and plotted against their time lag in Figs. 1–3. The cases varied greatly in size, time lag and character. We should not forget that these were particular, unique R&D projects that in many dimensions were widely divergent. In addition, over the entire period between the first cases and the present, the size of the market increased enormously: in Britain between 1736 and 2010 by more than two orders of magnitude, 135 times, and in the United States between 1790 and 2010 by more than three orders of magnitude, or about 3250 times (Officer and Williamson, 2010).

From Fig. 1 the large variation in project costs is immediately clear. Even if we cannot be extremely precise given the data quality, we can infer that the real costs of these R&D-projects spanned at least seven orders of magnitude, from 1/10,000 of the Empire State Building construction costs to 1000 ESI, the largest project—the Apollo project of the 1960s in this case—being one to ten million times the size of the smallest project—J. Frank Duryea's self-propelling road carriage from 1895. In few other areas in management and economics do we find such gigantic differences in scale, and Fig. 1 gives us a rare opportunity to quantify this degree of variation between the cases we studied. It is clear that R&D projects between c. 1750 and 2000 had at least this variation, and new cases can only extend the range, not reduce it.

It is also clear that eighteenth and early nineteenth century Britain was able to incur R&D-projects of substantial scale—the ship's clock, water frame, difference engine or self-acting mule having real GDP-deflated R&D costs that were of magnitude 2 (in the range of 0.001 ESI), similar to the R&D costs of car engines or soap in early twentieth century Britain, and to those of the DC-3, or the first catalytic cracking process in the United States. These early British cases, even though they are only a handful, reject the notion that pre-twentieth century society was unable to incur large-scale R&D projects.

Using our second measure, expressing R&D-costs as GDP-share (Fig. 2) rather than using the GDP-deflated ESI index, increases the relative importance of the early British cases. Compared to the size of the economy, the development of the ship's clock was a truly gigantic project, of an order of magnitude comparable to the R&D costs of the Manhattan project, the Houdry or fluid catalytic processes, or the development of the DC-8. One thus could say that the ship's clock was the eighteenth century's Manhattan project. The water frame came close to this scale as well. The spinning jenny, with far more modest development costs, was comparable to the development of the hoisting machine in 1880, the Linde liquefied air process of 1895, the tube tank catalytic cracking process of 1918, the DC-1/DC-2 of 1932 or the development and testing of recombinant growth hormone in 1982. The Babbage difference engine and the self-acting mule of the 1820s were comparable, as share of GDP, to DuPont's purchase of the Claude process in 1924, the development of titanium in 1946, that of Dacron in 1948, and Apple Computer's pre-IPO costs in the late 1970s. It is also clear from Fig. 1 that until at least the 1950s, British firms were able to carry out large scale research projects that were broadly similar in size to many large U.S. R&D projects.

Looking at the time lags (Fig. 3) it is clear that few positive time lags were larger than ten years, and few negative time lags (for prizes or patent purchases or R&D-in-process purchases) shorter than minus ten years. Of the positive time lag projects, very few projects had a long time lag and low costs. Of the negative time lag projects, very few had a long negative time lag, and very few had very high costs. Most cases were within two adjacent areas: between zero and five years in the range of 1/1000 to 1 ESI, or between five and ten years in the range of 1/100 to 1 ESI. It is also clear that the most costly projects did not have the longest duration. Babbage's difference engine seems to be an outlier with twenty years of development. It is also clear that the Manhattan project and the Apollo project were the biggest cases of their time. In Fig. 3, obviously lower and higher bounds can be drawn in which we find most of the cases.

### 3.3 The sensitivity of R&D outlays to cash flow

It is clear from the above that firms needed cash to carry out R&D: with very limited possibilities for collateral, real uncertainty, long multi-stage time lags, and information asymmetries, the market for the financing of R&D-projects was highly imperfect. This is not unrelated to the pecking order theory of corporate finance introduced by Myers and Majluf (1984), who argue that because of adverse selection, financers will demand higher returns on certain kinds of projects. External finance will thus be more expensive on these projects and a pecking order will emerge that effectively ranks financing alternatives in an order descending in the degree to which they enable managers to exploit investment opportunities: internal funds, then high-priority debt, lower priority debt, and finally equity (see also Triantis, 2000). R&D appears to be an extreme case because of the severe information asymmetries, the absence of collateral, the time lag and uncertainty.

This perspective is corroborated by present-day empirical evidence on the sensitivity of R&D-outlays to firms’ cash flow. If cash and financing constraints are important we would expect that R&D outlays are very sensitive to cash flow. If this were not the case, we could reject our hypothesis.

Although studies vary, they generally find that R&D-outlays are far more sensitive to changes in cash flow for smaller enterprises than for large enterprises (Table 6) (Himmelberg and Petersen, 1994; Ughetto, 2008).^12^ Estimates for the cash-flow elasticity of R&D vary from 0.4 to 0.8 between several studies, suggesting that a one percent increase in free cash flow would lead roughly speaking to a 0.6 percent increase in R&D (Bloch, 2005; Hall, 2002).

Brown and Petersen (2009) show that since 1970 the cash-flow elasticity of physical investment has declined sharply, perhaps because of better functioning capital markets, while the cash-flow elasticity of R&D outlays has remained. Vernon (2004) finds that after controlling for endogeneity large pharmaceutical firms’ R&D was still sensitive to cash flow, but that the sensitivity, 0.22, was lower than values from other studies (see also Grabowski and Vernon, 2000; Schroth and Szalay, 2010). Brown et al. (2009) even find that the 1990s R&D boom, mainly in internet-related technologies, can be largely explained by finance supply shifts that increased the cash available to young firms.

Despite the varying findings and the measurement difficulties all this evidence points to the notion that firms needed cash, not capital to finance R&D-projects. Despite the five obstacles to obtaining research finance, they were generally able to finance projects. In the next section we are going to review some organisational forms and institutional instruments that firms used to finance R&D.

## 4 The institutional evolution of the allocation of cash for R&D

From the historical evidence above it is clear that aggregate R&D-outlays grew faster than GDP-growth for a long time, and that particular R&D-projects involving large scale outlays have existed at least since the mid-eighteenth century. Despite the financing obstacles, from early on firms were able to incur large amounts of R&D outlays for large and uncertain projects, resulting in a phenomenal growth of aggregate cash outlays on R&D.

This finding is not unrelated to other work on organisations and institutions. Ostrom (1990), for example, finds that although classical economic theory predicts that common pool resources such as fishing grounds, commons or water supply will be depleted without government intervention, communities developed many different ways to govern the common pool resources. In practice few were depleted.^13^ Likewise, Chandler's historical masterwork (1962) showed convincingly that organisations did matter in economic processes. In Williamson's (1981) words ‘after Chandler, nobody could argue anymore that organisations did not matter’. Williamson (1985) shows how firms are able to solve transaction problems that would be problematic if carried out through a market.

**Fig. 4:**
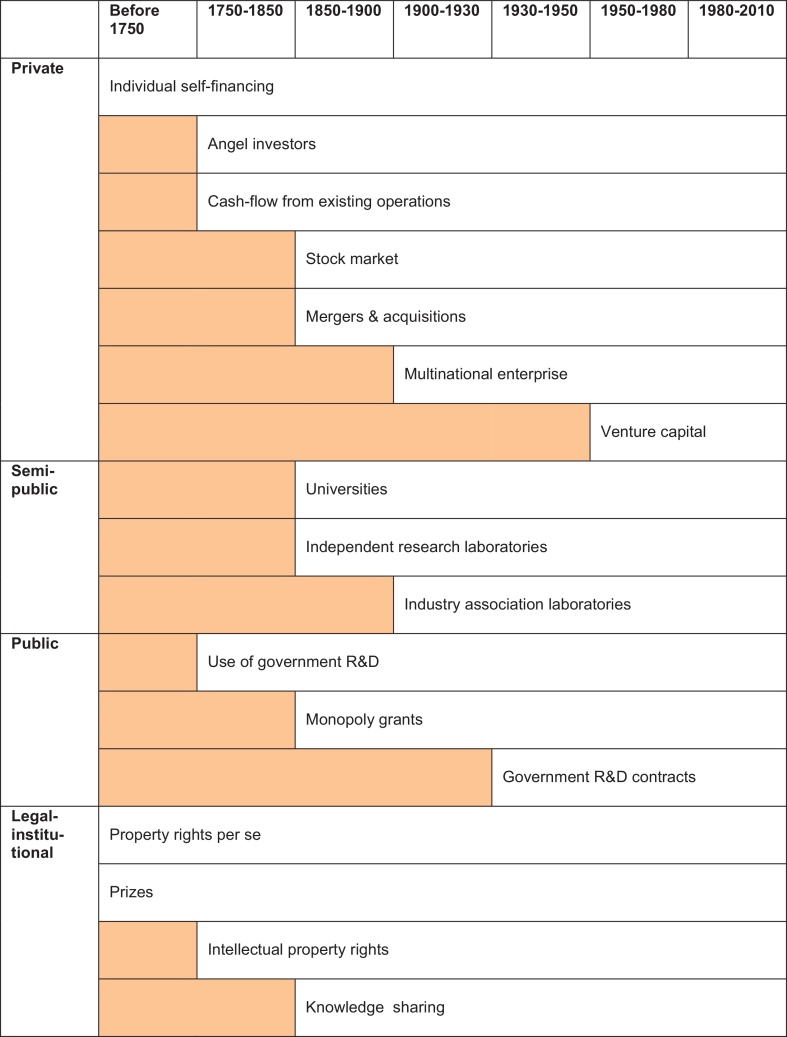
Historical emergence of institutional solutions to the R&D-financing problem. *Note*: this is an informal and broad periodisation. The period refers to the period when the solution became widely adopted for the financing of R&D, not to the period when the underlying organisational form or institutional instrument first appeared. Source: see text.

Likewise, we note that in practice firms have been resourceful and creative in finding solutions to the R&D-financing problem. We will argue that over time a series of cash allocation devices emerged that allowed firms to accumulate cash and sink it into R&D-projects. In Fig. 4 the most important of these are shown in the period when they rose to dominance. These solutions enabled arbitrage to take place in cash for R&D projects, through individuals, such as angel investors, through organisations, such as multinationals, and through institutions, such as venture capital.

The cash allocation devices we discuss below can be divided into several different, overlapping ways. They can be divided into devices depending on internal and those depending on external financing. They can be divided according to whether they can provide small-scale or large-scale financing. They can be divided according the stage in the R&D process they most easily finance: early stage or later stage R&D (Branscomb and Auerswald, 2001, 2002). They can also be divided into whether they use free cash flow or not. And they can be divided into private, semi-public, public and legal-institutional devices. Given that the latter division most closely relates to our research question, we will use it to guide our discussion, keeping the other comparative dimensions in mind, and revisiting them in the comparative discussion at the end.

We will discuss subsequently, in chronological order, the following private institutions: individual self-financing, angel investors, free cash flow from existing operations, the stock market, mergers and acquisitions, multinationals, venture capital, and R&D financing organisations; the following semi-public institutions: universities, independent labs and industry association labs; the following public institutions: government R&D, monopoly grants and government R&D-contracts; and, finally, the following legal-institutional instruments: property right, prizes, intellectual property rights, and knowledge sharing. This series is not exhaustive: we have restricted ourselves to the major institutional solutions. Although they emerged gradually over time, the R&D-financing solutions were not mutually exclusive. In the late twentieth century an R&D project, for example, could use different devices to get cash for different stages of R&D. It could start, for example, as a project in a university, followed by self-financing by individuals, followed by an angel investment, then venture capital, an IPO and a merger. As normal as the staged use of these devises may seem today, with some R&D projects going through several of them, they emerged historically, and to some extent in the order that they are used today. By briefly discussing these devices in succession, we aim to show how each addressed some aspect of the R&D financing problem, resulting in broad spectrum of financing options available today.

Many organisational solutions had several different purposes and solved various different challenges simultaneously. We are not arguing that each of these devices had as main purpose the financing of R&D—some clearly had other important purposes, but we do argue that many devices could be and were used for mitigating the R&D-financing problem.

### 4.1 Private institutions

The purest, simplest and probably oldest solution to the R&D-financing problem is obviously self-financing by individuals. Experimenting at one's own cost might even take place within animal species. It solves the sunk costs and information asymmetry obstacles, and given that there is no pressing need for profits, the cost of uncertainty and long time-lags are probably felt less. The latter, of course, also might reduce the incentive to push for commercial innovation. Many examples exist of gentleman-scientists who made massive contributions to science rather than focus on commercial application.

Charles Darwin and Henry Cavendish are well-known examples. A post-war British exponent is Peter Mitchell who built his own research lab at his country mansion and developed the chemiosmotic hypothesis, for which he won the Nobel Prize (Kealey, 1996, p. 75). Striking cases are also Edmund Cartwright who funded the development of the power loom from his own fortune, J.B. Lawes who together with G.H. Gilbert invented superphosphate at his Rothamsted farm lab in the 1820s, and Eugène Houdry who used his family fortune to develop the Houdry catalytic cracking process during the 1920s (Allen, 2009; Kealey, 1996; Freeman and Soete, 1997). The latter case also shows how self-financing is limited by the size of one's fortune, as Houdry eventually had to form a joint-venture with two oil firms to pay for development costs.

Another cash allocation device was the use of the angel investor, an investor who provided cash at a very early stage under flexible conditions. The angel investor allowed the innovator to incur sunk costs by providing cash. The time-lag became also less pressing because, contrary to bank loans, no regular interest payments were required. Uncertainty remained. The angel investor did bear all three problems of sunk costs, uncertainty and long time lags, but by definition they were independently wealthy and could miss the cash. Adverse selection and moral hazard remained, but generally angel investors mitigated this in three ways. Sometimes they financed family projects, where family ties decreased the angel's monitoring costs and increased the innovator's cost of opportunism. Sometimes they had made their fortune in related industries so were knowledgeable about the innovator's field. Often they operated in informal networks exchanging information and monitoring jointly with other angels. Examples of the latter were probably the Lunar Society during the Industrial Revolution, of which many leading industrialists were member. A late nineteenth century French example was a group of families around Lyon that had made their fortune in silk and textiles and which supported firms in new industries. By providing easy cash they bankrolled Charles Pathé’s audacious entry into the phonograph and motion picture business, his company becoming the largest film producer–distributor in the world before 1914.

Sometimes entrepreneurs that made their fortune in one new industry were happy to put cash in other new and uncertain projects, with the full knowledge they might not get it back. At other times entrepreneurs had a more direct interest in the project they backed, and were also likely to contribute knowledge and contacts. James Watt, for example, was initially backed by the owner of a drowned mine that could not be saved with existing pumps. Likewise, Hargreaves’ development of the spinning jenny was bankrolled by a textile industrialist, who paid him a wage, board and lodging and all the costs of the prototype and assistants. This must have been a significant amount, and at that time it was not at all clear that the venture would pay off. Hargreaves’ presence on his backer's estate helped mitigate moral hazard by allowing continuous monitoring. A famous American angel investor was the author Mark Twain, of St Louis, who was fascinated by technology and a close friend of Nikola Tesla. Twain almost bankrupted himself by putting a sum of $190,000 (about $4m in 2005 dollars, or $173m in GDP-share) in a failed type-setting machine. He also funded various other projects, such as the creation of one-handed grape-shears, perpetual calendars and a cloth made from peat (Lewis, 2011).

Taking cash flow out of existing operations is a tried and tested method to finance R&D. It differs from self-financing by individuals in that existing operations deliver a cash flow that can be sunk in R&D within the same business. The German chemical firms of the later nineteenth century, for example, started to sink cash flow from their dyestuff business into pharmaceutical laboratories which eventually grew into large and profitable pharmaceutical divisions (Beer, 1958; Liebenau, 1988). Likewise, ICI, the British chemical conglomerate, started sinking some cash flow into a pharmaceutical division from the mid-1930s. Only after twenty years did it start to make some profit, and only after thirty years did it become very profitable, with the introduction of several new types of drugs such as corticosteroids and beta blockers (Owen, 1999). Nowadays high technology firms such as Apple, Cisco, Google, Facebook and Amazon hold large cash piles in part to finance R&D and the acquisition of patent portfolios and R&D-firms (see introduction, above).

With cash flow financing the monies are generally written off immediately, so that the financing problems associated with sunk costs, uncertainty and the time lag are mitigated, as no costs are carried in the accounts that need to be written off if a project fails. The funding method assumes, of course, a cash flow that is large and long-lasting enough to sustain R&D projects. Studies showing the present-day sensitivity of R&D outlays to cash flows have been discussed above.

The adoption of modern incorporation laws during the nineteenth century (Harris, 2000) constituted a step change in the cash allocation possibilities for R&D. The corporation mitigated sunk costs because they were shared by many investors, because dispersed shareholders could diversify, because shares were transferable so shareholders were not tied to the R&D-project for its duration, and, finally, because limited liability shareholders were less concerned about uncertain high-sunk cost projects with a small but not insignificant likelihood to bankrupt the firm.

The corporation mitigated the problems caused by time lags, first, by offering transferable shares, second, by keeping cash locked-in because it did not face the potential call on its assets that partnerships faced upon exit of a partner, and, third, by the fact that corporations could survive beyond the life of its managers, owners and employees.

Adverse selection and moral hazard were alleviated by doing research in-house and setting up in-house R&D-labs. Schumpeter (1942, p. 96) noted that “the first thing a modern concern does as soon as it feels that it can afford it is to establish a research department.” The latter was not only important for generating inventions, but also for being able to find, screen and buy outside inventions, to be able to prepare the acquisition of other companies, and to generally assist in anticipating future industry development (Simon, 1993; Nicholas, 2010).

Finally, corporations’ delegated control allowed for the governance of free cash flow, so that it stayed inside the corporation and could be used for things such as R&D, rather than be claimed by shareholders. Kamien and Schwartz (1982, p. 28) suggest that if stockholders accept normal stock returns on the presumption that management has superior knowledge, extraordinary profits will allow firms to finance R&D in an uninhibited, flexible manner. Some studies, however, find shareholders myopic, showing how share prices generally fall when R&D-outlays rise, even if in the past such increases led to high returns. Goodacre and Tonks (1995, pp. 317–318), for example, find a negative effect of R&D-outlays on share prices, using a complete data-set based on forced disclosure and thus preventing sample selection bias for the public announcement of ‘good’ R&D-projects. They also note the myopic incentive of managers to cut R&D-expenditure, as it will immediately increase profits. Likewise, Munari et al. (2010) find a greater pressure towards the reduction of R&D in market-based governance systems such as in Britain and the United States (see also O'Sullivan, 2000; Tylecote and Ramirez, 2006). Other studies, however, do not find a negative effect of corporate governance on R&D spending, making the evidence mixed. Meulbroek et al. (1990), for example, find that U.S.’ firms R&D/sales ratios decline after implementing takeover defences, and Hall and Hall (1993) do not find evidence of shareholders myopia towards R&D (see also Hall, 1994). The various studies might highlight different sides of the same coin, especially since corporate governance is hard to measure unambiguously. Triantis (2000), for example, argues that, partially because of the pecking order of financing alternatives, too little financial slack prevents the firm from exploiting profitable investment opportunities, while too much slack encourages managerial misbehaviour and exacerbates agency problems. Christensen (2008) explains how over-use of financial tools leads to underspending on R&D, for example by erroneously comparing projects against a status quo that will persist in the absence of R&D.

The nineteenth German chemical firms did not actually make a return on investment calculation when founding research labs (Liebenau, 1988, p. 118). Carl Bosch, the CEO of IG Farben explained that “[R&D] is not there to give big profits to our shareholders. Our guide and our duty is to work for those who come after us to establish the processes on which they will work” (Hayes, 1987; von Tunzelmann, 1995). Usually big projects required ten years of research, yielded ten years of substantial returns and another ten years of sagging returns, according to Bosch. ICI held similar views on its fledgling pharmaceutical business.

Business history encompasses many cases in which shareholder activism leads to curtailment of R&D spending. Curtiss-Wright, for example, a leading American aircraft firm in the 1930s, planned a post-war R&D budget of $36m ($290m in 2005 dollars and $2.0bn as GDP-share). After a campaign of key shareholders the R&D budget was slashed and partially paid out as dividend. Subsequently the CW-20 plane failed and Curtiss-Wright had to leave airframe manufacturing to become a major component maker (Sutton, 1998, p. 431). The case appears to corroborate Christensen's (2008) critique on the overuse of financial tools for R&D-planning.

Another way to obtain cash was an initial public offering (IPO) on a stock market. The modern stock market developed during the nineteenth century in tandem with the new incorporation laws. In Victorian Britain and in the United States listing requirements were lax and early-stage firms were floated in industries such as cars, cinema, music, planes and electricity. Although many focused on the application of proven innovations, such as the railway companies, bicycle manufacturers and cinema operators, a few concerned research into unproven technologies (Michie, 1981, 1988).

As regulation became stronger it became more difficult for new industries to get cash through IPOs. After 1945 the modern venture capital industry emerged, which grew faster when, in 1971, the NASDAQ opened. This exchange had lower listing requirements, which were even further relaxed in the 1980s. A symbiosis emerged in which the flotation option stimulated venture capital because profitable exit was now possible without positive cash flow. An IPO also released continuous information about how others valuated the venture. If anybody knew more about its true value it should show in price movements and short selling.

Although it has been almost impossible to get cash through an IPO exclusively for early-stage R&D, from the perspective of the firm, a stock market flotation solved the issue of sunk costs, as no regular interest payments were needed. It probably also mitigated the pressure of the time lag, especially if governance was rather imperfect. For investors, the stock market partially mitigated the sunk costs problem and the time lag, since shares were now readily tradable and an investor could exit any time. It also reduced uncertainty because in theory the stock price contained perfect information, containing all relevant persons’ views on the expected pay-off of the firms sunk R&D-outlays.

Another way to obtain cash for R&D was through mergers and acquisitions, referred to simply as ‘mergers’ hereafter. We restrict ourselves here to mergers by large firms. We will ignore the buying of small technology firms by big firms; our main focus is on the merger as a device for increasing cash flow. Mergers mitigated the sunk costs problem when the merged firm had larger absolute cash flows, allowing more, larger scale and longer-term R&D-projects, even if the R&D/sales ratio actually fell. On the supply side mergers allowed more R&D to be done as there was a larger market share with a larger cash flow, while at the same time on the demand side a larger market share meant that R&D once it was finished, could be rolled out more quickly and enjoy shorter pay-back periods.

The trusts in the late nineteenth century United States did not get many cash flow advantages, because member companies remained separate entities. In the wake of the antitrust acts, when firms needed to merge if they wanted to set prices legally, the cash flow to merged firms increased and a sharp rise in R&D-outlays was initiated (Nicholas, 2003). A clear historical example of M&As leading to more cash flow and then to more R&D is the chemical industry in the first half of the twentieth century. DuPont in the United States and IG Farben in Germany became gigantic firms with enormous R&D budgets and long time horizons. Escalating R&D costs persuaded the individual firms to form IG Farben in 1925 (Freeman, 1963). In 1928, Standard Oil of New Jersey paid an unprecedented $35m ($330m in 2005 dollars, and $4.3bn as 2005 GDP-share) for the rights to IG Farben's patent portfolio outside of Germany (Table 5). In Britain, ICI, within four years after its 1926 merger quadrupled its R&D budget to £1m, about a quarter of all R&D done in Britain and three quarters of that in the chemical industry, rising to £1.4m in 1939, about 17 percent of all British R&D (Hannah, 1983, p. 113). As noted above, ICI had a very long-term horizon and was willing, for example, to sink cash in pharmaceuticals for over twenty years without seeing any profit.

Having many divisions, these firms could take a longer term perspective, as they could sustain negative cash flow in one division for some time, allowing longer payback periods. Absent a market for R&D-cash, capital markets could only do this very imperfectly on their own, and firms with multiple business units were needed. Cassiman et al. (2005), for example, find a strong increase in R&D if merging firms have complementary technologies.

Another example of an industry in which mergers were important for R&D-financing was civil aviation, which, starting from relatively modest development costs in the 1920s, became one of the most R&D-intensive industries. Aircraft manufacturer Douglas, for example, struggled under the burden of escalating R&D-spending (Table 5) and the rapid expansion of production of its best-selling but underpriced DC-9 plane. Sutton (1998, pp. 447–448) argues that when Douglas reported profits of only $4m in the first quarter of 1966, the stock market began to look askance at the company's recent accounting change under which it stopped writing off R&D-outlays as they occurred, but entered them in the accounts as an asset under deferred charges, thus enhancing apparent profitability. The company's stock price collapsed by 75 percent, and the company needed a cash injection of $400m. It was only saved by a merger with McDonnell. Thirty years later R&D-costs had become so high that even the merged company could not afford them anymore and merged in its turn with Boeing.

Mergers mitigated technical uncertainty by joining the R&D of two firms, and strategic uncertainty by taking out another innovator that could launch competing products. Market uncertainty was alleviated by having a larger market share, making the launching of new products easier, and profit uncertainty was reduced by having a larger cash flow from the enlarged market share to pay for fixed costs. This probably also reduced the time lag between the start of sales and actual profits.

Adverse selection and moral hazard were mitigated by not using external finance to fund R&D, but by bringing another, external source of cash flow inside the firm through merger. The acquirer could mitigate adverse selection by approaching targets itself, rather than by responding to overtures by firms wanting to be bought. Acquisitions were also a way to buy a bundle of R&D projects that there difficult to separate from each other and from the firm-specific knowledge of the target firm.

Sometimes the target firm could be capitalised on the acquirer's balance sheet and written off in several years, meaning that the target's R&D became capitalised (Lev, 1999). Whether the acquired R&D was immediately written off or capitalised, an approximate momentary valuation of it had taken place because of the market transaction, much like a solar eclipse revealing information about the sun. While in the United States since 1974 expenditure on R&D has to be written off immediately, several other countries allow firms to capitalise acquired and/or their own R&D (Lev and Sougiannis, 1996). Only in some industries, however, was R&D a large part of the acquired firm.

R&D by a multinational enterprise is of course a special form of using cash flow from existing operations to finance R&D. It merits separate treatment as the multinational provided a mechanism that helped this cash to cross borders. The hypothesis that multinationals exist to arbitrate in capital has been questioned at least since the 1960s (Jones, 2000). However, in the case of R&D, instead of arbitrating in capital, multinationals arbitrated in R&D-cash, for which no market existed and which had no law of one price, and therefore could only cross borders through a particular institution, such as the multinational firm.

Modern multinational enterprises emerged during the late nineteenth century and became prominent during the first half of the twentieth century. They undoubtedly expanded abroad for many other reasons than R&D (Jones, 2000). Yet, as Buckley and Casson (1991) argue, after 1945 firm-specific knowledge became a key driver of the expansion of the multinational enterprise, which became “an international intelligence system for the acquisition and collection of basic knowledge relevant to R&D”.

Between the 1960s and the 1990s, for example, electronic and pharmaceutical multinationals’ Foreign Direct Investment (FDI) existed for eighty percent of greenfield investments, in the majority of cases exploiting their firm-specific knowledge (Kuemmerle, 1999). More recently, Unn and Cuervo-Cazurra (2008) found that subsidiaries of multinationals used less external R&D than domestic firms, because they could use their parent's R&D, and Gorodnichenko and Schnitzer (2010) found that local firms in transition economies such as those in Eastern Europe were less innovative and productive than foreign-owned firms, and attributed this to their difficulty in attracting capital and presumably also cash for innovation (see also Nicholas, 2011).

The multinational had particular knowledge combined with a pile of cash, and this allowed it to sink outlays that domestic firms could not sink because they lacked either the knowledge or the cash. The multinationals’ foreign cash flow mitigated the sunk costs problem, its knowledge technical uncertainty, its international distribution network market and profit uncertainty. If it bought stakes in existing firms, its technical knowledge helped to mitigate adverse selection and moral hazard, while its international market knowledge helped it to better valuate the target than domestic firms.^14^

A major form for the financing of early-stage R&D is venture capital. A large literature exists on venture capital and here we will only discuss it briefly in terms of our obstacles and in a historical perspective. Early forms of financing resembling venture capital existed already in Britain during the Industrial Revolution, the venture capitalists being called ‘projectors’ (Brunt, 2006; Allen, 2009). A century later, in Victorian Britain, many venture capital-like investments were placed in firms in new industries that were subsequently floated on the stock exchange (Michie, 1981, 1988). Modern venture capital emerged in the 1940s in the United States, and its history has been well-documented elsewhere (Lerner, 2009). Its evolution was dependent on large-scale government cash outlays on defence and health R&D. American Research and Development Corporation, set up and run by Georges F. Doriot in 1946, is widely considered to be one of the first venture capital firms. It made a huge profit from an investment in Digital Equipment Corporation. ARD was followed by many other firms, and several regulatory changes helped spur a boom in venture capital. From 1971 technology companies could be floated on the NASDAQ stock exchange, reducing the time lag in which investments were locked-in, and in 1981 NASDAQ listing requirements were further relaxed. In the late 1970s jurisprudence allowed pension funds and endowments to invest in venture capital. Relaxed Californian labour law allowed employees to rapidly switch between different firms in the same industry: they could leave a firm and work at a related firm the next week. Unlike their East Coast counterparts, Californian employers were unable to prevent this because of the unenforceability of post-employment covenants not to compete (Saxenian, 1994; Gilson, 1999; Bankman and Gilson, 1999). Since the 1970s, large corporations also spun off research into separate ventures. General Electric was one of the first firms to have a dedicated programme for this. In the 1980s the Bayh-Dole Act (1980) and the Federal Technology Transfer Act (1986) were introduced that facilitated the commercial exploitation of inventions from universities and government labs (Mowery, 2009).

Already in the 1920s Knight (1921, p. 333) mentioned the possibility of an entrepreneur specialising in setting up many new ventures and others investing in him; the investors can assess his track record in organising new ventures. Knight called this ‘capitalisation of the entrepreneurial function’ and also discussed how, with more ventures in one enterprise, errors could cancel each other out.

From the innovator's perspective, venture capital mitigated the sunk costs and time lag problems by providing long-term cash in steps upon the achievement of technical milestones. From the capitalist's perspective, the sunk cost problem was largely solved through five different factors. Initial discovery costs were often borne by universities or government labs, with the venture capitalists only picking the survivors. Staged financing limited the cash sunk until the next decision point. Unlike corporate R&D-labs individual ventures were clearly separated and could be closed down quickly and smoothly. Finally, often a life insurance policy was taken out on key scientists.

The investor's uncertainty problem was partially solved by technical milestones, enabling the frequent reassessment of a project's prospects, and by betting on industries through portfolio investing, rather than on individual ventures. Patents protected against imitation and enabled funding, because they allowed the revelation of technical details. The time lag problem was mitigated by the possibility to float ventures early on the stock market, or to sell it to large corporations. Adverse selection was alleviated by having scientists on the financer's board, by knowledge gained through funding multiple projects in one technical area and by investing in consortia with other venture capital firms with knowledgeable managers. Picking only the successful projects from universities and government labs that generated large amounts of inventions without a profit motive, also reduced adverse selection. Moral hazard was reduced by giving key scientists equity stakes and stock options, having seats on the board of start-ups and by regular site visits. In this way, venture capital firms could provide an incentive system for key scientists, that large corporations found difficult to provide (Zucker et al., 1998).

A financing method related to venture capital was that of the external research and development financing organisation (RDFO). Firms with a low marginal tax rate that lacked a sufficient cash flow or a cash pile to finance R&D from, put a specific, separable part of their research in a RDFO, in which outside investors with high marginal tax rates then invested, with the research in progress and patents as collateral (Beatty et al., 1995). The immediate tax deductibility of R&D was thus sold to investors, who valued it more, though Beatty et al. (1995) caution that taxes were likely not the only reason for the use of RDFOs. RDFO-type organisations were established after 1974 and RDFOs became widely used between 1980 and 1986, when over 150 were formed, raising over $1 billion per year at their peak (Beatty et al., 1995). They were often used by cash-constrained venture capital backed technology firms. Beatty et al. find that transaction costs and investor's concerns about adverse selection and moral hazard made the cost for the R&D-firm about two to three times that of a ‘seasoned equity offering’, and therefore RDFOs were mainly used by firms that were seriously cash-constrained. In 1982, Genentech, for example, raised $50 million by putting the further development and testing of manufacturing processes and alternative delivery systems for human growth hormone and gamma interferon in a separate entity underwritten by outside investors (Beatty et al., 1995, p. 416).

### 4.2 Semi-public and public institutions

The private institutions discussed above were embedded in other layers of semi-public and public institutions, as well as in legal-institutional instruments.^15^ The major semi-public institution that helped firms finance R&D was the university. In mid-nineteenth century Britain, several industrialists helped fund universities that included technical subjects, and many older universities also embraced those subjects more fully (Sanderson, 1972b). Likewise, from the mid-nineteenth century most German chemical firms used nearby university professors under consulting contracts. Many new products emerged from these, and their students staffed the growing corporate R&D-labs. Similarly, in the United States in the interwar period, new pharmaceutical firms would locate close to universities (Furman and MacGarvie, 2007).

Universities mitigated the sunk costs problem by bearing the costs of the ‘misses’, leaving the hits for firms to exploit. By bearing the fixed costs of a large existing research infrastructure they reduced the size of the fixed costs firms needed to incur. Uncertainty for firms was mitigated if university staff had already screened out unpromising leads, and when firms consulted university staff, they were assured by the hiring and publishing criteria in academia that they passed a minimum quality threshold. The university also shortened time lags, by performing the earliest-stage R&D, so that the clock only started ticking when a firm licensed it and began to sink cash. As noted in the discussion of venture capital above, adverse selection in the licensing of university innovations was probably low as they were non-profit institutions, and often firms approached universities rather than vice versa. In the late twentieth century universities sometimes may have become more proactive in pursuing revenues from scientific advances that emerged in the university, but the university organisations as a whole remained generally not-for-profit organisations heavily dependent on donations and endowments, and the set of scientific projects from which promising technologies were selected for commercial licensing was probably not strongly affected by commercial motivations (Mansfield, 1991, 1998).

Another semi-public institution was the industry association lab, where sunk costs were shared across an industry, so reducing strategic uncertainty. They became prevalent in interwar Britain (Sanderson, 1972a; Edgerton and Horrocks, 1994). Private independent research labs fulfilled a slightly similar function and had been in existence since at least the late nineteenth century. A prime example was the Edison Lab, which besides working on its own inventions, also had many contracts with outside firms (Dodgson and Gann, 2010, p. 92).

Besides private and semi-public ways to raise cash for R&D, public institutions were also important. The major ones were government laboratories, government R&D-contracts awarded to private firms, and state monopolies. Direct research by government laboratories helped firms to finance R&D in a way broadly similar to exploiting university research. Agricultural, defence and health R&D-labs have all been important. The U.S. National Institutes of Health, for example, have generated many inventions that were developed further by companies, such as the first HIV-medicine.

Another public institution was the government funding of R&D carried out by firms. Before the Second World War direct funding of R&D-projects by governments was less common. In the U.S. aircraft industry, for example, the Department of Defence would ask manufacturers to show off their prototypes on an airstrip at a certain date, and then chose one model to be manufactured (O'Sullivan, 2007). In interwar Britain, the government subsidised laboratories set up by industry associations, and financed several research-intensive programmes, such as the airship programme between 1924 and 1930 (Edgerton and Horrocks, 1994, p. 225).

After 1945, governments increasingly started to fund R&D directly through contracting. Alic (2013) provides a detailed historical study of U.S. defence contracting after 1945, showing how R&D became one of the largest components. Government R&D-contracts mitigated sunk costs, especially with cost-plus contracts, as the government paid for them, they lowered strategic uncertainty as it was usually known if the government contracted with other firms as well, and it reduced market uncertainty, as the single buyer had signalled it was interested in the innovation. The time-lag was also less pressing as there was already a positive cash flow in the R&D-phase through government payments. However, the firm did need to overcome adverse selection problems and had to find a way to signal to the government that it was a reliable contractor to carry out the R&D. A potential solution was for the government to approach the firm rather than vice versa. The firm might also submit to regular monitoring to overcome moral hazard.

Another public institution was the awarding of a monopoly by the state—often, but not always to a firm that was state-owned. An early example was the awarding of a monopoly to postal and telegraph services in Britain in the late nineteenth century. A prime modern example was the telecom industry in Europe and North America during most of the twentieth century. Many firms maintained large central laboratories, the most famous of which was Bell Labs, which spawned several Nobel Laureates. The labs often lacked a clear profit-motive and legitimacy to maximise revenue. Under pressure from anti-trust suits, Bell Labs, for example, licensed its technology for a minimal fee.

Monopolies alleviated the sunk cost problem, as the monopolist could set prices to ensure a certain amount of cash flow. It also reduced strategic, market and profit uncertainty by the absence of competitors. Time lags became also less pressing, for better or for worse, by the absence of competitors and the steady, guaranteed cash flow that could pay for R&D. Given the monopoly, external financers might even willingly finance the R&D.

### 4.3 Legal-institutional instruments

Other devices that helped firms finance R&D were legal-institutional solutions such as property rights per se, prizes, patents and knowledge sharing. The awarding of innovation prizes had been a tried and tested method. An iconic prize was the English Board of Longitude Prize (1716). For the innovator, a prize did not mitigate the sunk costs problem, but it did reduce profit uncertainty because of the guaranteed pay-off. However, a prize increased strategic uncertainty by encouraging more firms to enter the race, with a second innovator often not receiving anything. The time lag also became more pressing, as competitive pressure was high and prizes often carried a deadline.

From the prize-financer's perspective, the sunk costs problem was fully solved, because cash only needed to be paid upon a working innovation, and in theory the innovation could even function as collateral for external finance: it is conceivable that a bank would agree to lend the funds for the prize once it had to be awarded, given that the underlying, now proven, innovation could be expected to yield future revenues. Technical, strategic and market uncertainty were largely borne by the prize contestants. Profit uncertainty might have been higher, as given the many entrants and their ownership of partial property rights it was unclear whether and how the innovator could profit. Usually the prize was awarded by a government or private non-profit organisation for an innovation of public benefit, on the condition that it was placed in the public domain. The inventor of celluloid, for example, did not claim a prize because it meant giving away the intellectual property rights. Because of the ex-post character, the prize also perfectly solved adverse selection and moral hazard for its financers.

A social disadvantage was the risk of overspending on a particular R&D-trajectory. The $10m Ansari X-Prize for the first private human space flight, for example, resulted in total R&D outlays by all contestants of $100m. Brunt et al. (2012) have identified a multitude of prizes for agricultural innovations in nineteenth century Britain and find that they fostered innovation. Recently firms have also started to use prizes as a way to finance their R&D. A website, for example, offers firms to post their scientific problem and the award they will pay for the first working solution, with the website as guarantor to inventors.^16^ There are, of course, obvious disadvantages to this technique.

Another well-known legal-institutional mechanism was the patent. Patents mitigated the innovator's sunk costs problem slightly, because an unsuccessful R&D-project might still yield patents that could be sold or used to block a competitor. Patents reduced strategic, market and profit uncertainty by increasing imitation costs, and they made the time lag less pressing by delaying imitation. They also helped innovators to reduce the adverse selection problem as they could reveal more when they talked to external financers.

Another legal-institutional institution was knowledge sharing. Nineteenth century shipyards, for example, sometimes agreed to offer to each other all innovations they developed (MacLeod, 1999), and in the interwar period several large knowledge-sharing arrangements were signed, such as the famous patents and processes agreement between ICI and DuPont (Hannah, 1983, p. 117). Knowledge sharing agreements often gave access to innovations that had yet to be made at the moment of signing.

### 4.4 Comparing the institutions

Putting all the solutions to the R&D-financing problem we discussed in one diagram (Table 7) shows various R&D-cash allocation solutions that emerged at different points in history and, through survival, now all coexist together, offering the substantial menu of solutions that characterises today's society. Besides the private/public classification which we have used as the main ordering principle of the various cash allocation solutions, they also can be classified, of course, according to whether the finance is internal or external, whether projects are smaller or larger and whether they are in an earlier or a later stage (Table 7).

From the innovator's perspective the institutions that mitigated all five obstacles were, in chronological order, intellectual property rights, government R&D and venture capital. From the financer's perspective, institutions mitigating all obstacles were prizes and venture capital. Clearly venture capital is the only institution that mitigated all obstacles for both parties, and perhaps this explains its current popularity in financing particular kinds of R&D. Venture capital, was, of course, dependent on the rise of semi-public and public R&D and intellectual property rights.

Institutions mitigating fewest of the obstacles were, for innovators, independent research labs, closely followed by self-financing and prizes. For the financers they were government R&D contracts (where the government is considered the financer), followed at some distance by angel investors, and those followed again by property rights per se and the stock market.^17^ Prizes and government R&D-contracts were the most asymmetric institutions. Prizes mitigated all obstacles for the prize-financer, but left sunk costs and the time lag unresolved for the innovator, while government R&D-contracts mitigated all obstacles for the innovator but none for the financer (the government).

The obstacles that were easiest to mitigate with some institutional solution were, for innovators, sunk costs and adverse selection, and, for financers, uncertainty. The high score of uncertainty mitigation is partially due to intellectual property rights safeguarding value capture, and stock markets/IPO's delivering continuous information on the aggregate valuation of a project. The most difficult to mitigate obstacles were, for innovators, the time lag and, for financers, the time lag, adverse selection and moral hazard. The fact that the time lag is the hardest to mitigate obstacle for both parties, reflects the observations of Schumpeter, Keynes and Hicks that the time lag and the roundaboutness of the production process is one of the central features of capitalism, and absent from static equilibrium models.

## 5 Conclusion

We have examined the pattern of aggregate R&D spending in the very long run, since c. 1750, as well as how firms were able to incur large R&D outlays on particular, highly uncertain projects, in the face of substantial financing obstacles, following the historical case study approach of Chandler (1962) and the analytical-historical approaches of North (1981) and Williamson (1985). The particular R&D-project has been the key unit of analysis, and this is what makes this paper distinctive from studies focusing on aggregate firm outlays, national outlays or general non-financial aspects of R&D.

We have noted how the financing of R&D historically was made difficult by five challenges: the fact that R&D was characterised by sunk costs, real uncertainty, long and open-ended time lags between outlays and pay-offs, adverse selection, and moral hazard. The implication of these challenges was that firms needed cash, not capital, for R&D-projects. This has been corroborated by our review of studies on contemporary R&D-financing which in many instances concluded that firms’ R&D expenditures were highly sensitive to cash flow.

We found that the long-run pattern of R&D spending, both in Britain and the United States, revealed sharp growth, often several times faster than GDP-growth, from the turn of the century to the 1970s, with subsequent growth being equal to or lower than GDP-growth. We also highlighted how little is known about aggregate R&D-expenditure before 1900.

For case studies of particular R&D-projects, we introduced three methods to measure project costs. Besides using GDP-deflated costs and costs relative to GDP, we introduced the Empire State Index, which uses the GDP-deflated construction costs of the Empire State Building as a comparator. We found that even long before 1900 some R&D-projects had a non-insignificant scale: eighteenth century projects such as the ship's clock, the spinning jenny and the water frame all required substantial outlays. We also noted the enormous variation in costs and time lags of R&D projects: the costs varied between as much as seven orders of magnitude, meaning that the largest project in our case set was in the order of one to ten million times bigger than the smallest project. The time lag varied from about minus twenty to about plus twenty years. For catalytic cracking, aircraft and chemicals we also found some further evidence of the low-hanging fruit hypothesis—that R&D-outlays were increasing disproportionately along a technological trajectory. We also observed that despite the smaller British home market, British firms were able to engage in R&D-projects that were on a similar scale to major U.S. projects, such as ICI's wartime nuclear research, its terylene project and Beecham's semisynthetic antibiotics project. This is consistent with the findings of Edgerton and Horrocks (1994) and nuances Mowery and Rosenberg's (1989) account that British R&D was substantially behind that of the United States before 1940.

The historical facts that aggregate R&D-outlays grew faster than GDP-growth for a long time, and that particular large-scale R&D-projects have existed at least since the mid-eighteenth century, suggest that, despite the challenges to the financing of R&D, firms were able to incur R&D outlays for large and uncertain projects.

This finding is not unrelated to other work on institutions. Ostrom (1990), for example, finds that although classical economic theory predicts that common pool resources such as fishing grounds, commons or water supply will be depleted without government intervention, communities developed many different ways to govern their common pool resources, and that in practice few were depleted. Likewise, we noticed how firms and societies in practice proved creative and resourceful in finding solutions to what in theory should be an insurmountable financing problem.

We reviewed a series of cash allocation mechanisms that they developed for this purpose over time and that allowed them to sink ever larger amounts of cash in R&D-projects. We argue that these solutions to R&D-financing often served many other purposes, but that they also allowed firms to finance R&D, and increasingly so after 1945. Private institutions we reviewed included self-financing by individuals, angel investors, free cash flow from existing operations, IPOs, mergers, multinational organisations, venture capital and R&D financing organisations. Semi-public and public institutions included universities, industry association labs, government labs, government R&D-contracts and state-sanctioned monopolies. Legal-institutional instruments we reviewed included property rights per se, prizes, patents and knowledge sharing. We found that the only institution that mitigated all five obstacles for financer and innovator alike was venture capital. The most asymmetric solutions were prizes and government contracts: the former left important challenges for the innovator, while the latter mitigated few obstacles for the financer (the government).

We need to see these solutions in the context of a sharp jump in the size of the national market, by more than two orders of magnitude in Britain between 1736 and the present, and by more than three orders of magnitude in the United States between 1790 and today, in the context of new technologies that increased the effectiveness of R&D such as the periodic table, systematic soil sampling, the integrated circuit, or DNA-sequencing (Moser, 2012), and, finally, in the context of large public spending increases on R&D through which the pubic sector bore part of the sunk costs and minimum project outlays. Further research could explore ways to arrive at an estimate of aggregate R&D expenditure before 1900, and establish whether the history of R&D in other high-growth countries, such as Germany and Japan, fitted the British and American pattern.

The main implications of our research are, first, that cash is essential for financing R&D-projects: especially in times of financial crises, when the cash supply tends to dry up, policymakers might think about how to stimulate R&D. Second, a flexible legal framework is conducive for organisational experimentation and so allows firms to develop new organisational and contractual arrangements to finance R&D. Some argue that venture capital is relatively important in the United States, Britain and Israel at least partially because the common law in those countries affords substantial legal and organisational flexibility (Gilson, 2001). Third, institutional flexibility is important in order to have a law-making and regulation process that can adjust the legal framework and so allows for changes over time that can be conducive for R&D. In the United States, for example, institutional flexibility allowed relaxed Californian labour laws, the founding of the NASDAQ, the loosening of pension fund investment regulation, the relaxation of NASDAQ listing requirements, the Bayh-Doyle Act and many other laws and regulations that stimulated outlays on R&D. Fourth, by taking the project as unit of analysis and looking at the scale of particular historical cases, we hope that with the Empire State Index, we have provided an easy, intuitive comparative tool for policy makers, firms and academics to get a grip on the relative size of past R&D projects and long-run trends.

## Figures and Tables

**Table 1:** Selected generic solutions to the R&D-financing problem, by obstacle mitigated.

	Obstacle	Solutions
Inherent factors	Sunk costs	Staged funding
		Mile stones
		Write-offs
		Options
		Government funding
	Nested uncertainty	Options
		Patents
		Collusion
		Joint R&D
	Time lags	Largest amounts last
		Green lights
		IPO
		Annual write-offs

Transactional factors	Adverse selection	Who initiates (M&A)
		Scientists on board financer
		Personal links / social control
	Moral hazard	Board seats
		Company visits
		Large equity stakes top scientists & managers

*Note:* The solutions are not mutually exclusive, they were often used simultaneously. The solutions mentioned are examples; they do not form an exhaustive set.

**Table 2:** Taxonomy of successive types of uncertainty of an R&D project's outcome.

Type of uncertainty	Mitigated by	Increased by
Technical uncertainty	Advances in science and technology	Decreasing returns within a technological trajectory
	New techniques that increase effectiveness of R&D (e.g. periodic table, DNA sequencing)	
	Longer time lags	
	Option approach	

Strategic uncertainty	Competitive intelligence	Competition policy
	Product development announcements	Prizes
	Oligopoly	
	Joint R&D; M&A; collusion	

Market uncertainty	Market research	Luxury products with high income elasticities
	Shorter time lags	
	Prizes	

Profit uncertainty	Adequate business models	Unstable government policies and regulation
	Intellectual property rights	Unstable tax regimes
	Prizes	Piracy/imitation

**Table 3:** Growth rates of real R&D-expenditure in Britain and the United States, c. 1910–2008.

Type	Period	Growth rate (%/yr)		gR&D/gGDP	Source
		R&D-exp.	GDP		
*UK*					
All R&D	c. 1910–1938	4.7	1.0	4.7	Sanderson (1972a)
All R&D	1938–1945	18.2	2.4	7.7	Saul (1979)
All R&D	1945–1961	13.0	2.1	6.1	Saul (1979)
All R&D	1961–1969	3.3	3.0	1.1	Saul (1979)
All R&D	1964–1998	1.7	2.3	0.7	von Tunzelmann (2004)
Business R&D	1964–1998	1.8	2.3	0.7	von Tunzelmann (2004)
HE R&D	1964–1998	5.8	2.3	2.5	von Tunzelmann (2004)

*US*					
Business scientists	1921–1940	12.9	2.9	4.4	Mowery and Rosenberg (1989)
Business R&D	1930–1940	1.7	0.5	3.5	Mowery and Rosenberg (1989)
All R&D	1941–1963	11.0	4.0	2.8	Mansfield (1968)
All R&D	1953–2008	5.5	3.2	1.7	Nicholas (2011)
All R&D	1970–1999	3.4	3.2	1.0	von Tunzelmann (2004)

*Notes:* R&D refers to real R&D, deflated using the same deflators as for GDP, except for 'Business scientists'. GDP refers to real GDP deflated using Officer's (2011) and Johnston and Williamson's (2011) GDP-deflators. gR&D/gGDP refers to the R&D growth rate over the GDP growth rate. A value of 4.7, for example means that R&D-expenditure grew 4.7 times as fast as GDP. 'Business scientists' refers to the growth rate of scientists employed in corporate R&D labs and is uses for 1921–1940 instead of real R&D-expenditure growth, as that data is not available. HE R&D refers to R&D by higher education institutions.

**Table 4:** Selected cases of completed R&D-projects and their direct costs and mode of financing, Britain 1736–1957.

Year	Innovator	Innovation	Direct cost					Time lag	Category
			GDP-deflated	Opportunity costs		Empire State Index			
			(£ of 2005)	(% of GDP)	(£m 2005)	(ESI)	(Magnitude)	(years)	
1736	Harrison	Ship's clock	1,762,824	0.01730	217	0.0041	2	−20	Prize by Board of Longitude
1767	Hargreaves	Spinning jenny	134,824	0.00117	15	0.0003	1	3.5	Angel-rel. ind.
1768	Richard Arkwright	Waterframe	1,229,302	0.01021	128	0.0029	2	6	Angel-family; then projectors/VC
1823	Charles Babbage	Difference Engine	1,550,312	0.00375	47	0.0036	2	20	Government contract
1825	Roberts/Sharp	Self-acting mule	1,027,103	0.00248	31	0.0024	2	4	Self-financing; cash flow
1883	Priestman Brothers	Oil engine	4,233,365	0.00313	39	0.0098	2	11	Cash flow
1885	Cuthbert Heath	New insurance policies	700,000	0.00057	7	0.0016	2	3	Angel-family
1903	Napier	Car engine plant	2,990,826	0.00167	21	0.0069	2	3	Angel-family; angel-rel. ind.
1904	Lever Brothers	Soap mass manufacturing	2,432,432	0.00133	17	0.0056	2	2	Angel-unspecified; cash flow
1904	Courtaulds	Artificial silk	17,117,117	0.00934	117	0.0397	3	4	IPO; cash flow; divestments
1909	Louis Bleriot	Crossing Channel by plane	90,090	0.00005	1	0.0002	1	−0.5	Prize by Daily Mail newspaper
1919	Alcock/Brown	Transatlantic flight <72 h	381,679	0.00018	2	0.0009	1	−6	Prize by Daily Mail newspaper
1924	Vickers/Air Ministry	Airship programme	18,636,364	0.00891	112	0.0432	3	6	Govt. contract; direct govt. R&D
1941	ICI	Nuclear research	56,100,000	0.01707	214	0.1302	4	3	Government contract
1941	Calico Printers/ICI	Terylene	99,255,583	0.03340	419	0.2303	4	9	Cash flow
1952	Pilkington	Float-glass process	74,766,355	0.02053	257	0.1735	4	6	Cash flow
1957	Beecham	Semi-synthetic antibiotics	148,367,953	0.03483	437	0.3442	4	9	Cash flow

*Notes:* Year is the year that the R&D started, except for prizes, which show the year the prize was awarded. 1885, 1903 and 1904 are estimates based on the historical literature. Costs are direct historical cash outlays on R&D as documented in the sources and have not been discounted into one net present value using the time lags. Real direct costs have been calculated using the UK GDP-deflator from Officer (2011) for the mid-year in the project lifespan. Opportunity costs in £m are as percentage of 2005 GDP. Please note that costs are not precisely comparable. Sometimes development is included, sometimes not, and sometimes building of pilot plants is included, such as in the case of the waterframe and soap. Costs in this table should only be used to get an idea of the order of magnitude of R&D-expenditures, in the absence of systematic long-run project data, and not as exact and fully comparable figures. For the spinning jenny, Allen's (2009) estimate of direct costs has been doubled to account for Hargreaves’ opportunity costs and board and lodging received. The time lag has been estimated from the sources and should be taken as a ball park indication, especially for the 1885, 1903 and 1904 cases. For the airship programme and the nuclear research the time lag is simply the length of the research programme. For the ship's clock Harrison's first successful test has been taken as year, as he received numerous different payments, the first being close to that year. Angel-rel. ind.: an angel investor from an industry related to the innovator's industry. VC: Venture capital. Govt.: Government. Empire State Index (ESI): expresses the projects costs as fraction of the GDP-deflated construction costs of the Empire State Building (1931) in New York (see text). Magnitude: shows the order of magnitude of the Empire State Index, with 1 being the lowest observed order, which is between 1/10,000 and 1/1000 of the Empire State Building, and 7 being the highest observed order, which is between 100 and 1000 Empire State Buildings.

**Table 5:** Selected cases of completed R&D-projects and their direct costs and mode of financing, United States, 1875–1999.

Year	Innovator	Innovation	Direct cost					Time lag	Category
			GDP-deflated	Opportunity costs		Empire state Index			
			($ of 2005)	(% of GDP)	($m 2005)	(ESI)	(Magnitude)	(years)	
*Chemicals*									
1895	Brush/Carl von Linde	Liquefying air	4,863,813	0.00104	131	0.0109	3	10	Self-financing/cash flow
1924	DuPont	US rights to Claude process	26,641,294	0.00322	406	0.0595	3	1	Cash flow; patent collateral
1924	DuPont	Moisture-proof cellophane	369,159	0.00004	6	0.0008	1	3	Cash flow
1939	DuPont	Cellophane process improvements	165,162,455	0.00821	1036	0.3690	4	11	Cash flow
1946	DuPont	Titanium	61,475,410	0.00306	387	0.1374	4	7	Cash flow
1948	DuPont	Dacron	40,701,315	0.00181	229	0.0909	3	8	Cash flow

*Oil/catalytic cracking*									
1909	Standard Oil of Indiana	Catalytic cracking	1,476,726	0.00027	34	0.0033	2	4	Cash flow
1917	Universal Oil Products	Flow cracking process	53,191,489	0.00766	967	0.1188	4	5	Angel-rel.; cash flow unrel. firm
1918	Oil firm	Tube and tank cracking process	5,479,452	0.00082	103	0.0122	3	5	Unknown
1925	Houdry Process Corporation	Houdry catalytic cracking	135,970,334	0.01873	2365	0.3038	4	12	Self-finance; cash flow rel. firms
1929	Standard Oil of New Jersey	Purchase IG Farben patent portfolio	329,877,474	0.03376	4262	0.7370	4	0	Cash flow; patent collateral
1935	Houdry Process Corporation	TCC/Houdriflow process	13,387,660	0.00125	158	0.0299	3	8	Cash flow
1938	Consortium of six oil firms	Fluid catalytic cracking process	172,612,198	0.01479	1867	0.3856	4	3	Cash flow

*Aircraft*									
1927	Lockheed	First streamlined aircraft	238,322	0.00003	3	0.0005	1	1	Cash flow
1932	Douglas	DC-1/DC-2	5,804,041	0.00081	102	0.0130	3	2	Cash flow
1936	Douglas	DC-3	3,537,736	0.00041	52	0.0079	2	1	Cash flow
1952	Douglas	DC-8	652,680,653	0.02560	3231	1.4582	5	6	Cash flow; government subsidy
1952	Boeing	B707	186,480,186	0.00731	923	0.4166	4	6	Cash flow; joint with military version
1959	Douglas	Electra turboprop plane	408,942,203	0.01480	1869	0.9136	4	1	Cash flow
1964	Boeing	Boeing 747	3,662,109,375	0.09524	12,022	8.1817	5	4	Cash flow
1982	Hypothetical (est. by Boeing)	“Large commercial jet”	8,121,277,748	0.13833	17,462	18.1441	6	7	—

*Other innovations*									
1875	Unknown	Mechanical substitute for horses	180,505	0.00012	15	0.0004	1	−3	Prize by Wisconsin legislature
1880	Alexander E.	Brown Hoisting machine	1,851,852	0.00097	122	0.0041	2	2	Angel-family
1895	J. Frank Duryea	Self-propelling road carriages	108,696	0.00003	4	0.0002	1	−0.3	Prize by Chicago Herald Tribune
1908	Glenn Curtiss	Fly a plane for 1 km	40,850	0.00001	1	0.0001	0	−0.3	Prize
1912	A Canadian company	Acq. S.A. Baker's car heater patent	2,469,136	0.00043	54	0.0055	2	0	Cash flow; patent collateral
1920	Westinghouse Electric	Acq. radio patents from E. Armstrong	2,723,735	0.00040	50	0.0061	2	0	Cash flow; patent collateral; univ.
1930	RCA	Television	73,702,830	0.00852	1076	0.1647	4	9	Cash flow
1931	Comparative non-R&D example	Empire State Building	447,598,253	0.05361	6767	1.0000	5	1	Bank financing
1941	US Government	Manhattan project R&D	648,148,148	0.03185	4020	1.4481	5	4	Direct government spending
1942	US Government	Manhattan project pilot plants	17,870,370,370	0.87806	110,838	39.9250	6	3	Direct government spending
1961	NASA	Manned moonlanding	170,000,000,000	4.42098	558,060	379.8049	7	8	US Government
1961	NASA	Apollo launch vehicle engine devpt.	4367,075,665	0.12873	16,250	9.7567	5	5	US Government
1972	Cray Research	Supercomputer	28,007,175	0.00057	72	0.0626	3	4	Venture capital; founders
1976	Genentech	Genetic sequencing technology	115,233,090	0.00197	248	0.2575	4	5	Venture capital; founders
1977	Apple Computer	Home computer	9,357,861	0.00016	20	0.0209	3	4	Venture capital; founders
1979	Seagate	Disc drives	2,431,414	0.00004	5	0.0054	2	2	Venture capital; founders
1982	Lotus Development	Spreadsheet software	8,604,945	0.00015	19	0.0192	3	1.5	Venture capital; founders
1982	Genentech	H. growth hormone / gamma interferon	83,654,007	0.00127	161	0.1869	4	5	RDFO funding (excubation of finance)
1983	Ovation Technologies	Spreadsheet software	10,416,667	0.00017	21	0.0233	3	1	Venture capital; founders
1987	Multi-firm R&D consortium	New microchips	138,504,155	0.00172	218	0.3094	4	7	Government contract
1996	Burt Rutan	Privately-built spacecraft	10,332,713	0.00008	11	0.0231	3	−8	Prize; angel-unrel. ind. (Paul Allen)
1999	Google	Improved search technology	57,623,603	0.00053	67	0.1287	4	4	Venture capital; founders

*Notes*: Year is the year that the R&D started, except for prizes, which show the year the prize was awarded. For some cases estimates had to be made based on the historical literature. Costs are direct historical cash outlays on R&D as documented in the sources and have not been discounted into one net present value using the time lags. Real direct costs have been calculated using the US GDP-deflator from Johnston and Williamson (2011) for the mid-year in the project lifespan. Opportunity costs in $m are as percentage of 2005 GDP. The costs are not precisely comparable; see the note under Table 4. For the cases of Cray Research, Apple Computer, Seagate and Lotus Development, the costs are the pre-IPO invested cash by founders and venture capitalists. The time lag has been estimated from the sources and should be taken as a ball park indication. Aircraft R&D-costs are very rough indicative costs, as civilian R&D was not always separable from military R&D (the Boeing 707 R&D was partially done for a military tanker version, for example), and because development expenditures were probably included to a different degree in different cases. Angel-rel. ind., an angel investor from an industry related to the innovator's industry. Univ.: University. Empire State Index (ESI): expresses the projects costs as fraction of the GDP-deflated construction costs of the Empire State Building (1931) in New York (see text). Magnitude: shows the order of magnitude on the Empire State Index, with 1 being the lowest observed order, which is between 1/10,000 and 1/1000 Empire State Building, and 7 being the highest observed order, which is between 100 and 1000 Empire State Buildings.

**Table 6:** Estimates of R&D elasticity and investment elasticity to cash flow from selected studies, 1974–2006.

Country	Period	Industry	Measured parameter	Elasticity	Source
US	1980–2001	Pharmaceuticals	Drug-price elasticity of R&D	0.6	Giacotto, Santerre and Vernon (2005)
Italy	1998–2003	Small Italian mfg. firms	Cash flow elasticity of R&D	Strongly positive	Ughetto (2008)
US	1983–1987	179 firms in high-tech industries	Cash flow elasticity of R&D	0.67	Himmelberg and Petersen (1994)
US	1983–1987	179 firms in high-tech industries	Cash flow elasticity phys. I.	0.82	Himmelberg and Petersen (1994)
US	1974–1994	Pharmaceuticals	Cash flow elasticity of R&D	0.22	Vernon (2004)
US	1974–1994	11 major drug firms	Cash flow elasticity of R&D	Strongly positive	Grabowski and Vernon (2000)
US	1970–2006	High tech firms	Cash flow elasticity of R&D	“Comparatively strong”	Brown and Petersen (2009)
US	1970–2006	High tech firms	Cash flow elasticity phys. I.	“Largely disappears”	Brown and Petersen (2009)
US	1990–2004	Young high-tech firms	Cash flow elasticity of R&D	“Significant effects”	Brown, Fazzari and Petersen (2009)
US	1990–2004	Mature high-tech firms	Cash flow elasticity of R&D	Insignificant	Brown, Fazzari and Petersen (2009)

*Note*: phys. I.: physical investment.

**Table 7:** The mitigation of R&D-financing obstacles by selected institutional cash allocation mechanisms, for the innovator and the external financer.

Institution	Since: circa	Example	Source	Scale	Stage	Obstacles to R&D-financing mitigated					
						Inherent			Transactional		Total number mitigated
			I/E	S/L	E/L	Sunk costs	Uncertainty	Time lag	Adverse selection	Moral hazard	
*Private*											
Self-financing	<1750	Power loom (1785)	Internal	Small	Mixed	1	–	–	1	1	3
Angel investors	1750/1850	Spinning jenny (1767)	External	Small	Earlier	1/–	–/–	1/–	1	1	4/2
Free cash flow	1750/1850	Oil engine (1883)	Internal	Large	All	1	–	1	1	1	4
Stock market/equity	1850/1900	Artificial silk (1904)	External	Large	Later	1/1	–/1	1/1	1	1	4/5
Mergers & acquisitions	1850/1900	ICI/Nylon (1926/40)	Ext./Int.	Large	All	1	1	–	1	1	4
Multinational enterprises	1900/1930	Viagra/sildenafil (1989)	Internal	Large	All	1	1	–	1	1	4
Venture capital	1950/1980	Gene sequencing (1970s)	External	Small	Earlier	1/1	1/1	1/1	1	1	5/5
RDFO	1980/2000	Genentech/hormone(1982)	External	Mixed	Later	1/–-	–/–	–/–	–	–	1/–
*Semi-public*											
Universities	1850/1900	Stanford (1950s -)	External	Mixed	Earlier	1	1	1	1	–	4
Independent labs	1850/1900	Edison/carbon light (1879)	Int./Ext.	Mixed	Earlier	1	1	–	–	–	2
Industry association labs	1900/1930	Agricultural innovations	External	Mixed	Earlier	1	1	–	1	1	4
Public											
Use of government R&D	1750/1850	Manhattan Project (1941)	External	Large	Earlier	1	1	1	1	–	4
Government R&D contracts	1930/1950	Apollo Project (1961)	External	Large	Earlier	1/–	1/–	1/–	1/–	1/–	5/0
Grant of indefinite legal monopoly	1930/1950	British postal and tele- Communications (1869)	Internal	Large	All	1	1	1	1	1	5
*Legal-institutional*											
Property rights per se	<1750	Largest telescope (c. 1800)	Int./Ext.	Mixed	Mixed	1/1	1/1	–/–	1	1	4/3
Prizes	<1750	Ship's clock (1736)	External	Mixed	Later	–/1	1/1	–/1	1	1	3/5
Intellectual property Rights	1750/1850	IG Farben portfolio transfer (1929)	Int./Ext.	Mixed	Mixed	1/1	1/1	1/1	1	1	5/4
Knowledge-sharing	1850/1900	Two shipbuilders (1888)	Ext./Int.	Mixed	Mixed	1	1	–	1	1	4

Total (no.) 18			9/14	11/15	16/11	17/5	13/5	9/4	16	14	69/21
Total (%) 100			50/78	61/83	89/61	94/63	72/63	50/50	89	78	77/53

*Notes*: within the four categories the cash allocation mechanisms are listed in broad chronological order. “Since” does not refer to an exact year but instead refers to the period in which the institution became widespread. The scale and stage for each institution have been assessed for the typical R&D-project in the respective category. “1” signifies that the institution mitigates the relevant obstacle; “–” signifies that it does not mitigate the relevant obstacle, where two values appear, the first reflect the innovator's perspective, the second the financer's perspective. RDFO: R&D Financing Organisation (see Beatty et al., 1995). For the independent research lab, the obstacles to the commissioner of the research are assessed. Internal financing, almost per definition, strongly mitigates the two transactional obstacles.

## Footnotes

1 Amounts rounded to the nearest billion. “Technology giants at war,” The Economist, 1 December 2012, p. 28. As percentage of annual revenue the cash piles were, respectively, 78, 9, 99 and 228 percent, and as percentage of the firms’ market value 22, 5, 21 and 18 percent.

2 Joseph Schumpeter, as quoted in McCraw (2006, p. 261).

3 For historical studies of the role of sunk costs in particular industries, see Bakker (2005).

4 This is mostly the case in the United States since 1974. Some other countries allow the capitalisation of some separable and later stage R&D, and firms can write these expenses off over several years; see Lev (1999).

5 Technical, market and profit uncertainty can be found back in Kamien and Schwartz (1982, pp. 109–110), who implicitly include strategic uncertainties under market uncertainties. See also Lazonick (1991) on productive and market uncertainty. Besides the project uncertainty this section focuses on, one can of course also distinguish more general uncertainties further removed from the direct environment of the R&D-project such as wars, natural disasters, depressions, financial crises or inflation.

6 Indivisibilities and inappropriability are the others. The first is partially dealt with under sunk costs, and we argue scale per se should not matter, because if markets for R&D-finance were perfect, firms should be able to undertake R&D-projects of almost any size. We group inappropriability under profit uncertainty here because it is a factor in whether a firm will be able to capture the profits of its innovation. Arrow does not explicitly address the problems of sunk costs and the time lag. See also Arrow (1983).

7 See the U.S. case studies in the next section.

8 See also Arrow (1962), for an early discussion of these as ‘the moral factor’.

9 See below.

10 In effect, the cash-constrained low marginal tax rate R&D firm sells the tax deductibility of its R&D to investors with high marginal tax rates (see Section 4.1 for a more detailed discussion).

11 For a discussion of expenditure per firm and historical British R&D expenditure in general see Sanderson (1972a), Saul (1979), and Edgerton and Horrocks (1994). For a historical introduction on the US situation see Mowery and Rosenberg (1989), and Lamoreaux and Sokoloff (2007).

12 Mulkay et al. (2001), using cash flow net of R&D outlays but gross of ordinary investment for large U.S. and French manufacturing firms between 1979 and 1993, find little difference between the sensitivity of their R&D and their ordinary investments to cash flow net of R&D outlays.

13 In many respects R&D is the obverse of common pool resources (CPR): applied R&D is partially excludable and non-diminishable (nonrivalrous) while CPR are non-excludable and non-diminishable. CPR also involve hardly any sunk costs, uncertainty, time lags, adverse selection and moral hazard. CPR already exist while R&D-projects need to be realised. Solutions to CPR-problems focus on preventing over-use, while solutions to R&D-problems tend to prevent underinvestment. The financing of CPR-exploitation is relatively easy, that of R&D relatively hard.

14 For a detailed historical case study on the role of knowledge and sunk costs in the evolution of multinationals see Bakker (2006).

15 On the multiple layers of institutional arrangements in which particular cases are embedded, see, for example, Ostrom (1990).

16 http://www.kaggle.com.

17 Alic (2013) provides an intriguing historical study of the inefficiencies in government contracting of U.S. defence R&D since 1945.
